# Detection of a Low Level and Heterogeneous B Cell Immune Response in Peripheral Blood of Acute Borreliosis Patients With High Throughput Sequencing

**DOI:** 10.3389/fimmu.2019.01105

**Published:** 2019-05-16

**Authors:** Josiane Kirpach, Alessia Colone, Jean-Philippe Bürckert, William J. Faison, Axel R. S. X. Dubois, Regina Sinner, Anna L. Reye, Claude P. Muller

**Affiliations:** Vaccinology and B Cell Immunology, Infectious Diseases Research Unit, Department of Infection and Immunity, Luxembourg Institute of Health, Esch-sur-Alzette, Luxembourg

**Keywords:** *Borrelia*, B cell repertoire, Lyme, Borreliosis, VlsE-C6, tetramer, multicolor flow cytometry, *in vitro* stimulation

## Abstract

The molecular diagnosis of acute Borreliosis is complicated and better strategies to improve the diagnostic processes are warranted. High Throughput Sequencing (HTS) of human B cell repertoires after e.g., Dengue virus infection or influenza vaccination revealed antigen-associated “CDR3 signatures” which may have the potential to support diagnosis in infectious diseases. The human B cell immune response to *Borrelia burgdorferi* sensu lato—the causative agent of Borreliosis—has mainly been studied at the antibody level, while less attention has been given to the cellular part of the humoral immune response. There are indications that *Borrelia* actively influence the B cell immune response and that it is therefore not directly comparable to responses induced by other infections. The main goal of this study was to identify B cell features that could be used to support diagnosis of Borreliosis. Therefore, we characterized the B cell immune response in these patients by combining multicolor flow cytometry, single *Borrelia-reactive* B cell receptor (BCR) sequencing, and B cell repertoire deep sequencing. Our phenotyping experiments showed, that there is no significant difference between B cell subpopulations of acute Borreliosis patients and controls. BCR sequences from individual epitope-reactive B cells had little in common between each other. HTS showed, however, a higher complementarity determining region 3 (CDR3) amino acid (aa) sequence overlap between samples from different timepoints in patients as compared to controls. This indicates, that HTS is sensitive enough to detect ongoing B cell immune responses in these patients. Although each individual's repertoire was dominated by rather unique clones, clustering of bulk BCR repertoire sequences revealed a higher overlap of IgG BCR repertoire sequences between acute patients than controls. Even if we have identified a few *Borrelia*-associated CDR3aa sequences, they seem to be rather unique for each patient and therefore not suitable as biomarkers.

## Introduction

Borreliosis, the most common tick transmitted disease in Europe and the United States, is caused by the *Borrelia burgdorferi* sensu lato bacterium or spirochete (short *Borrelia*). The antibody response to the pathogen has been studied extensively during the past decades, but the biology of the disease is still insufficiently understood. A number of serological tests are available (1–4) to support the diagnosis, but in the absence of the pathognomonic erythema migrans (EM) the diagnosis of Borreliosis remains often challenging (5–9).

Early studies of antibody kinetics in humans infected by *Borrelia* demonstrated a slow and heterogeneous response, which seemed to correlate with spirochete dissemination and onset of symptoms prior to therapy (10–12). IgG and IgM antibody titres can remain high for years and decline only slowly even after successful treatment (11–14). Thus, positive serologies even after resolution of the disease can complicate the diagnosis.

In Europe, the most important vector carrying and transmitting *Borrelia* pathogens is the *Ixodes ricinus* tick, while in America *Ixodes scapularis* and *Ixodes pacificus* are the main vectors (15). In nature, *I. ricinus* ticks, feed on a variety of hosts. In order for *Borrelia* to survive, they need to be transmitted not only from the feeding tick to the host, but also from the host to the next feeding tick. Because of this transmission cycle *Borrelia* had to adapt to different hosts and ticks, making them masters in modulating protein expression (8, 16–19). Many virulence determinantss are expressed in plasmids, which vary between strains (19, 20). Their expression determines clinical manifestations and disease progression (15). *Borrelia* species differentially regulate surface proteins to evade host immune responses (8, 16–19). Because of a greater diversity in *Borrelia* genospecies (21), the situation is even more complex in Europe than in North America (22). The epidemiology of tick bites and erythema migrans, indicates that individuals may be protected against one but not necessarily against other *Borrelia* strains (23). In line with this, low levels and heterogeneous B cell immune responses toward *Borrelia* have been described previously (10, 24–28). Mouse studies showed that reinfection even with the same *Borrelia* strain is possible, especially after antibiotics treatment (29). They showed furthermore, that both ticks (30, 31) and *Borrelia* (29, 32–36) actively influence the B cell immune response. Indeed, the tick seems to inhibit the local production of antibodies secreted by plasma cells, but not the formation of memory B cells (30, 31). For *Borrelia*, it was shown that during infection germinal center architectures were disrupted (32, 33). In addition, infected mice even failed to produce a robust protective antibody response to a co-administered influenza vaccine (29). Whether this is also the case in humans is not clear. Many of the above issues complicate studies and in particular the recruitment of patients.

The reactivity of patients' sera with *Borrelia* antigens has been used to identify *Borrelia* epitopes to improve serological tests or vaccine candidates (37–39). Despite their importance for diagnosis and protection, few studies have dissected the antibody repertoire in response to infection (40–43). Detailed analysis of patients' B cell repertoires by high throughput sequencing (HTS) revealed, that in some cases antigen-associated “signatures” with the potential to support diagnosis could be identified (e.g., for Dengue and influenza) (44–48). In the present study, we combined phenotypic analysis by multicolor flow cytometry with single cell BCR analysis and HTS of the B cell repertoires of recently/acutely infected individuals to analyse the peripheral B cell response to *Borrelia* and to identify CDR3 signatures of acute Borreliosis.

## Materials and Methods

### Study Participants

For the present study, 15 patients with erythema migrans diagnosed as acute Borreliosis have been recruited from Luxembourg. One donor (Lyme6) caught the infecting tick in Vienna (Austria). The B cell immune responses of acute patients was compared to both healthy donors and donors with a recent tick bite (Supplementary Table 1). The majority of acute Borreliosis patients and some of the controls were sampled at three timepoints within 1 month after diagnosis (T0: ~diagnosis, T1: 1 week after T0, T2: 4 weeks after T0). Patients were treated with antibiotics following the recommendations of their medical doctor. On average antibiotics treatment was started 4 ± 5 days before the first blood sample. Controls were age matched but included less female participants. Ticks of donors with a recent tick bite were confirmed to be negative for both *Borrelia* and *Rickettsia* DNA using an in-house PCR protocol (49). Additionally, tick bite donors that were included in the analysis of the BCR repertoire by HTS were seronegative for *Borrelia*. A previous study in Luxembourg (49) showed that with a mean infection rate of 11.3% for *Borrelia burgdorferi* sensu lato and 5.1% of *Rickettsia* those are the two most prevalent pathogens found in ticks from this country. In that same study (49), *Borrelia afzelii, Borrelia garinii, Borrelia burgdorferi* sensu stricto, *Borrelia valaisiana, Borrelia spielmanii*, and *Borrelia lusitaniae* were found in ticks. This study was approved by the competent ethical committee of Luxembourg (Comité National d'Éthique de Recherche, CNER). All donors signed an informed consent sheet. In addition a notification and authorization was submitted and accepted by the Commission nationale pour la protection des données (CNPD) in Luxembourg.

### Blood Draw and Freezing of PBMC Samples

PBMCs were obtained from 30 ml of heparinized (VACUETTE^®^ TUBE 9 ml NH Sodium Heparin, Greiner Bio-one) blood by Ficoll (GE Healthcare) gradient using Leucosep tubes (Greiner Bio-one) within <8 h after the blood drawing. PBMCs were frozen in freezing medium containing 10% DMSO (50) and stored in liquid N_2_. Serum was collected in Z serum sep clot activator tubes (VACUETTE) and stored in aliquots at −80°C.

### *Borrelia* Serology Testing

Commercial kits from Sekisui Virotech (*Borrelia afzelii* IgM ELISA, *Borrelia afzelii* + VlsE IgG Europe ELISA, *Borrelia* Europe Plus TpN17 LINE IgG Immunoblot, *Borrelia* Europe LINE IgM Immunoblot) were used to determine the serological status of participants. An in-house *Borrelia*-VlsE-C6 peptide ELISA was developed using biotinylated peptides (51) as antigen synthesized by EMC microcollections (Tübingen, Germany) with >90% purity: MKKDDQIAAAIALRGMAKDGKFAVK (*Borrelia bugdorferi s. s*. strain B31), MKKDDQIAAAMVLRGMAKDGQFALK (*Borrelia garinii* strain IP90) and MKKRNDNIAAAIVLRGVAKDGQFALK (*Borrelia afzelii* strain PT7). ELISA plates were coated overnight at 4°C with peptide in carbonate buffer (SIGMA). Between steps, plates were washed three times with washing buffer containing phosphate-buffered saline (Lonza) and 1% Tween-20 (Sigma). Plates were blocked for 2 h at RT with blocking buffer containing 1% bovine serum albumin (Sigma). Serum samples were diluted 1:100 and incubated for 2 h. Goat Anti-Human IgG-AP (SouthernBiotech) was added at 1:700 dilution and incubated for 90 min at RT in blocking buffer. Optical density was measured at 405 nm after incubation of the appropriate substrate (SIGMA) for 1 h at 37°C.

### Multicolor Flow Cytometry

PBMCs were stained immediately after thawing in FACS buffer (PBS with 0.1% NaN_3_ and 2% FBS; Lonza). Color compensations were done using a mix of cells and compensation beads (BD Biosciences). After incubation of the cells with antibodies on ice for 30 min, they were washed with FACS buffer. Hoechst (Invitrogen) live/dead marker was added shortly prior to measuring on a FACSAria SORP machine (BD Biosciences). The following antibodies were used in the panel: CD14-eFluor605NC, CD24-eF450, CD43-APC, CD23-APC-eFluor780 (eBioscience), IgD-BV421, CD19-BV605, IgG-PE (BD Pharmingen), CD10-BV510, CD138-BV711, CD27-PECF594 (BD Horizon), IgM-BV570, CD38-PerCP-Cy5.5, CD21-PE-Cy7, CD20-AF700 (BioLegend), CD5-FITC, CD3-PE-Dy647 (Immunotools).

### FACS Staining for Antigen-Specific Single Cell Sorting

To sort antigen-specific cells, tetramers were generated as described in Franz et al. (52). A mix of Neutravidin (Neutravidin DyLight650, Thermo Scientific) and biotinylated peptide was incubated in the dark on ice for 30 min. Aggregates were removed by high speed centrifugation. B cells from acute Borreliosis patients were stained for 30 min with a pool of the three peptide tetramers and washed twice with FACS buffer (PBS, 2% FBS). The following antibodies were used to distinguish the different memory B cell subpopulations and to gate out monocytes, T-cells and dead cells: CD14-FITC, CD3-FITC (Immunotools), IgD-BV421, CD27-PECF594 (BD Horizon), CD19-BV605 (BDPharmingen), and live/dead marker. CD20-Biotin (Immunotools) was used as a compensation control for Neutravidin. Single cells were sorted on a FACSAria SORP machine (BD Biosciences) into 96-well PCR plates (Eppendorf) containing 5μl of 0.5% PBS, 10 mM DTT (Invitrogen), and 5U Recombinant RNasin® Ribonuclease Inhibitor (Promega) per well (53). The plate holder of the sorter was kept at 4°C throughout the sorting procedure. Random, negative and tetramer positive CD19^+^CD27^+^CD14^−^, CD3^−^, Hoechst^−^ B cells were sorted into 96-well plates, which were immediately put on dry ice and stored at −80°C.

### Reverse Transcription for Single Cell PCR

Reverse transcription was performed in an Eppendorf vapo.protect thermal cycler. Nine microliters of reverse transcription buffer 1 containing 5 μl 2x FS buffer (Invitrogen), 3 μl of lysis buffer (5% Nonidet P-40/Igepal CA630, SIGMA) and 1 μl of random primers (150 ng/μl, Invitrogen) were added to each well containing a sorted cell. The first reverse transcription step was performed at 65°C for 10 min followed by 25°C for 3 min. The lysed cells were kept on ice for at least 1 min before pipetting 5.5 μl of the second mastermix containing 0.5 μl of 100 U SSIII reverse transcriptase (Invitrogen), 2 μl of 5x FS buffer (Invitrogen), 2 μl of 0.1M DTT (Invitrogen) and 1 μl of 2.5 mM dNTPs (Invitrogen). After mixing and a short spin, the plates were incubated at 37°C for 1 h followed by 70°C for 15 min to inactivate the enzyme.

### Generation of Heavy Chain Immunoglobulin PCR Products for Sanger Sequencing

Single cell PCRs were performed using the heavy chain primer (please refer to Supplementary Table 2) sets from Wang and Stollar (54) without the restriction sites (55, 56). First round and second round PCRs were performed in a total volume of 50 μl. For the first round PCR, a mastermix plate containing 5 μl 10x buffer, 2 μl 2.5 mM dNTP, 0.4 μM final concentration of each primer, and 0.25 μl HotStar Taq polymerase (QIAGEN) per well was prepared and 8 μl of cDNA mixture from the single cells was added. The PCR was performed in a UNO96 Thermal Cycler from VWR (denaturation and activation at 95°C 15 min, three cycles at 94°C 45 s, 45°C 45 s, 72°C 1 min, 40 cycles at 94°C 45 s, 50°C 45 s, 72°C 1 min, elongation at 72°C 10 min). Three microliters from the first round PCR were entered into the second round using the same mastermix with different primers (denaturation and activation at 95°C 15 min, 30 cycles at 94°C 45 s, 50°C 45 s, 72°C 1 min, elongation at 72°C 10 min). Wells without sorted cells served as negative controls for the PCR. PCR products were purified using Jetquick PCR Product Purification Spin kit (Genomed Gmbh) or in case of multiple bands QIAquick Gel Extraction kit (QIAGEN). Sanger sequencing was done in house.

### *In vitro* Single Cell Stimulation

The *in vitro* stimulation protocol (57) was kindly provided by Elisabetta Traggiai and John Lindner (Department of Mechanistic Immunology, Novartis Institutes for BioMedical Research, Basel, Switzerland). B cells were negatively selected from an aliquot of frozen PBMCs with the Easy Sep Human B cell enrichment cocktail kit (Stemcell Techonologies). The isolated B cells were then stained using the following antibodies: anti-CD3 PE, anti-CD14 PE (Immunotools), anti-CD16 PercPcy5.5 and anti-CD56 PecPcy5.5 (Biolegend), anti-CD20 Alexa Fuor 488 (Biolegend), anti-CD27 BV421, anti-IgD PE (BD Biosciences), anti-IgA and anti-IgM (Jackson Immuno Research). IgG expressing memory B cells were negatively selected on a FACSAria Sorp machine (BD Bioscences) by gating on CD3-CD14-CD16-CD56-CD27+CD20+IgD-IgA-IgM- B cells. The sorted cells were resuspended in supplemented RMPI 1640 medium and seeded in 384-well plates at 1 cell/well together with 2.5 × 10^5^/ml of irradiated CD40L EL-4-B5 cells (50 Gy) and incubated in a cocktail similar to Huang et al. (58) to stimulate the production of antibodies. Each plate contained 10 wells with only 2.5 × 10^5^/ml irradiated CD40L EL-4B 5 cells as negative control supernatants for the screening ELISAs. Cells were incubated at 37°C and 5% CO_2_. After 12–13 days, the supernatants were screened for antibody reactivity. Wells from one plate per donor were tested for antibody production using a commercial quantitative total IgG ELISA (Human IgG total Ready-SET-Go!® ELISA from eBioscience, Supplementary Figure 5). Specific antibodies were detected in 384-well plates using 2 μg/ml *Borrelia* VlsE-C6 peptide.

### Library Preparation and Deep Sequencing

RNA was extracted from frozen PBMCs with the AllPrep DNA/RNA extraction kit (QIAGEN) according to manufacturer's description. RNA yield was quantified by NanoDrop spectrophotometer. Next generation sequencing of the B cell repertoires of patients was performed on an in-house PGM IonTorrent (Life technologies). Deep sequencing libraries were prepared with an adapted version of the UID protocols described by Vollmers et al. (50) and Büerckert et al. (59) using BIOMED-2 V-gene primers (60). Primers (see Supplementary Table 2) were purchased from Eurogentec. Barcoded primers were purified by PAGE while standard SePOP desalted primers were used for second strand synthesis. Unless otherwise specified, PCR and reverse transcription reagents from Invitrogen were used and PCR reactions performed on a UNO96 Thermal Cycler (VWR). Stringent conditions were used to avoid cross sample contaminations. One microliter of 10 mM dNTP solution and 2 μl of 10 μM barcoded isotype primers were added to a 96-well PCR plate and 500 ng (for IgM)/1,000 ng (for IgG) RNA was added to each well and the total volume adjusted to 9 μl with RNAse/DNase free water (Gibco, Life Technologies). Reverse transcription was performed at 65°C 5 min, 55°C 80 min, 70°C 15 min. Eight microliters containing 4 μl First Strand Buffer, 2 μl DTT, 1 μl RNAseOUT, and 1 μl SSIII were added to each well at 55°C while still on the PCR machine. After the reverse transcription, 30 μl of second strand mix containing 10 μl Phusion HF buffer, 7 μl water, 10 μl 10 μM V-region primer mix, 1 μl 10 mM dNTP solution, 0.5 μl Phusion High-Fidelity DNA Polymerase (Bioke), and 1.5 μl DMSO was directly added to each reaction and second strand PCR performed (98°C 2 min, 63°C 2 min, 72°C 10 min). Then, two consecutive manual Agencourt® Ampure® PCR purification bead (Beckman Coulter) cleanups were done using 1:1 bead to sample ratio and otherwise adhering to the manufacturer's instructions. The double stranded DNA was eluted in 21 μl water of which 20 μl were added to a new PCR plate. Final PCR amplification was performed in a mastermix containing 10 μl Q5 buffer, 10 μl GC enhancer, 4.5 μl water, 2.5 μl 10 μM adapter primer mix, 2 μl 10 mM dNTPs, and 1 μl Q5^®^ Hot Start High-Fidelity DNA Polymerase (Bioke) at 98°C 5min, 19 to 20 cycles of 98°C 10 s, 65°C 20 s, 72°C 30 s, followed by a final elongation at 72°C 2 min. The obtained libraries were purified as before once using Agencourt® Ampure® PCR purification beads and the quantity determined on a 2100 Bioanalyzer Instrument (Agilent Technologies). Libraries were adjusted to equal concentrations using the amplicon peak of the expected range as measured by the 2100 Bioanalyzer (Agilent Technologies). A second Agencourt® Ampure® PCR purification bead cleanup of the pools was usually performed to eliminate primer dimers before quantifying the pool on the 2100 Bioanalyzer Instrument. The pools were sequenced using Ion PGM™ Template OT2 400 Kit, Ion PGM™ Sequencing 400 Kit and ION 318 CHIP KIT V2 (Life technologies) according to the manufacturer's instructions.

### Data Analysis

The raw data plots from the obtained Sanger sequences were analyzed and consensus sequences manually corrected by using SeqTrace (61). IMGT/V-QUEST (62) was used to characterize the different sequences in terms of V and J gene usage as well as for CDR3 region identification. Clustering of single cell sequences was done using the bcRep R package 1.3.4 developed by Bischof and Ibrahim (63). Since we observed non-specific binding of VlsE-C6 tetramers to a CLL clone identified in one of the donors, all the VlsE-C6 reactive sequences which clustered with those from CD5^+^ cells isolated from that same donor were excluded from data analysis. Kaluza Flow Cytometry Analysis Software was used for multicolor flow cytometry data analysis. Graphs and figures of this manuscript were made using Powerpoint and Graphpad Prism 5. Mean and Standard Error of the Mean are represented in the different graphs. Statistical significance is indicated as follows: ^****^: *p* < 0.0001; ^***^: *p* = 0.0001–0.001, ^**^: *p* = 0.001–0.01; ^*^: *p* = 0.01–0.05. Deep sequencing raw reads were processed following the pipeline described previously (59). Briefly, raw reads were demultiplexed by their MID barcode, the ssUID and primers clipped and stored in the sequence identifier. Only reads with QSC over 20 for more than 80% of the bases were considered for further processing. Reads were grouped into families according to their 16 nt ssUIDs. Consensus sequences were built for each ssUID family with more than 2 members using pagan-msa (64). Consensus sequences were collapsed to unique sequences and uploaded to IMGT/High V-QUEST (65) for selection of productive final consensus sequences, assignment of V, J, and CDR3 sequences as well as determination of V-gene similarity to germline genes. VDJtools (66) was used for analysis of V and J gene distributions as well as repertoire diversity. Clustering of sequences from deep sequencing data was performed using Change-O (67) and the Python script kindly provided by Galson et al. (44, 48, 68). Unless otherwise indicated, the Python script (44, 48, 68), was used to cluster HTS repertoire sequences of the same V-J combination allowing one mismatch for every six CDR3 amino acids (i.e., 83% CDR3 identity). The Python script (44, 48, 68) was also used to cluster single cell sequences according to different thresholds of CDR3aa similarities. For clustering of sequences with the Change-O pipeline (67), a clonal grouping threshold was determined for each individual donor with distance to nearest neighbor calculation (SHazaM R package). Briefly, length-normalized distance to nearest neighbor was calculated for each donor using a human Ig-specific single nucleotide model (hs1f). The local minimum of the resulting bimodal distribution of distance frequencies was determined using a custom-made R script and subsequently used to perform clonal grouping using the DefineClones python script of the Change-O (67) package.

### Phylogenetic Tree Analysis

Phylogenetic trees were generated using the one click approach from phylogeny.fr (69, 70). The germline sequences from phylogenetic trees in this study were determined manually by using the first V germline sequence determined by IMGT (62) until the V-D-J junction. As junction, the most frequent sequence within a clone was taken. The J gene was taken again from the identified germline sequence.

## Results

### Phenotyping of Peripheral Blood B Cells

**Figure 1:**
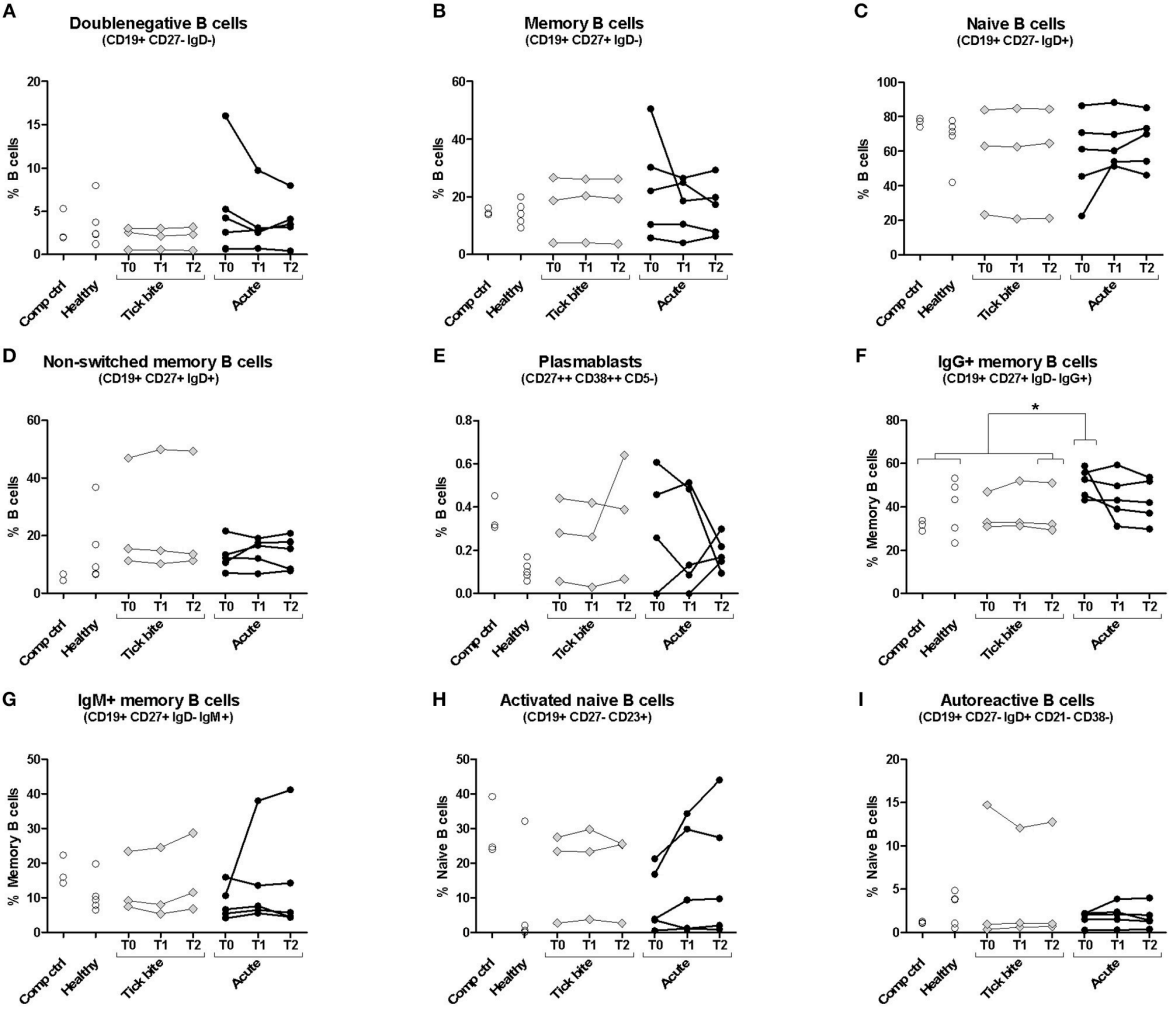
Peripheral blood B cell subset composition assessed with multicolor flow cytometry. Five erythema migrans patients (black circles, average age of group: 57, 60% female), and 9 controls (healthy: empty circles; tick bite: gray filled rhombi, average age of group: 60, 22% female) are shown. Two-tailed unpaired *t*-test showed no difference in age between both groups. Doublenegative B cell **(A)**, memory B cell **(B)**, naive B cell **(C)**, non-switched memory B cell **(D)**, plasmablast **(E)**, IgG+ memory B cell **(F)**, IgM+ memory B cell **(G)**, activated naive B cell **(H)** and autoreactive B cell **(I)** subpopulations were gated as described in Supplementary Figure 1. For color compensation, cells from one healthy donor (“Comp ctrl”) were combined with color compensation beads, stained and measured individually with each antibody. This donor sample was also analyzed with the antibody cocktail (multicolour control sample represented in this figure). “Comp ctrl”: Healthy donor sample used for compensation control and to assess reproducibility between the three experiments (each dot represents one experiment). “Healthy”: healthy individuals sampled at a single timepoint. “Tick bite” and “Acute”: Individuals with a recent tick bite or acute Borreliosis respectively sampled 3 times (T0: ~diagnosis, T1: 1 week after T0, T2: 4 weeks after T0) over 1 month. An unpaired two tailed t- test with 95% CI was used to compare single timepoints of the two groups: T0 for the acute patients (= closest to diagnosis and beginning of treatment); T2 for tick bite donors (= closest to healthy status quo ante and farthest away from the tick bite). For this analysis “Comp Ctrl,” “Healthy,” and “Tick bite” donors were grouped into a single “Control” group (for more information please consult Supplementary Figure 2).

We first investigated the human B cell response to *Borrelia* by multicolor flow cytometry (for gating strategy see Supplementary Figure 1). Peripheral B cell subset composition of five erythema migranspatients were compared with nine control samples, including six healthy donors and three donors with a recent tick bite (Figure 1). In most cases, B cell subset composition was relatively stable throughout the observation period (1 month), confirming also the reproducibility of our approach. The time curve in particular of the plasmablasts (Figure 1E) and the activated naïve B cells (Figure 1H) showed a tendency to be more variable in acute Borreliosis patients (mean maximum difference between timepoints for plasmablasts: 0.25% and activated naïve B cells; 10.01%) as compared to tick bite donors (0.16 and 2.47% respectively), but the difference between individual or aggregated timepoints of the three groups was not significant. The comparison of acute patients with controls revealed a significant increase in IgG expressing memory B cells at the timepoint of recruitment (Figure 1F, Supplementary Figure 2). Interestingly in one of the donors with a recent tick bite, we observed a particularly skewed peripheral B cell subset composition. This donor had highly expanded non-switched memory B cells (Figure 1D) as well as CD21^low^CD38^low^ autoreactive B cells (71) (Figure 1I) combined with relatively high levels of plasmablasts (Figure 1E).

### VlsE-C6 Epitope Tetramer Staining Indicates a Low Level and Heterogeneous *Borrelia*-Reactive Memory B Cell Immune Response in Acute Borreliosis

**Figure 2:**
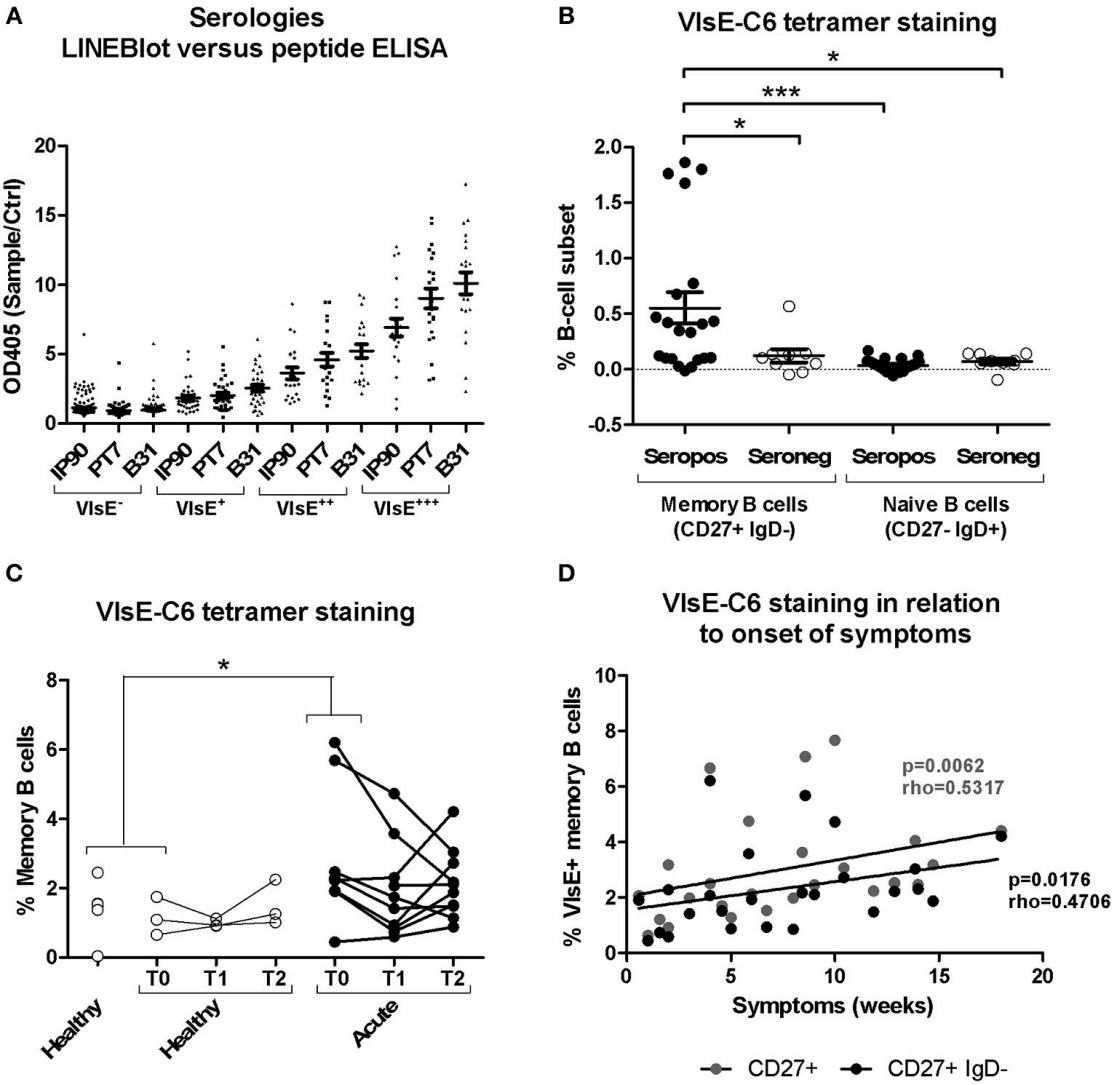
Isolation and characterization of *Borrelia* VlsE-C6-reactive memory B cells. **(A)** Sera (dilution 1:100) from 255 healthy donors with a high risk for tick bites and hence for *Borrelia* seroconversion were screened with both a commercial immunoblot (Sekizui Virotech) and our in-house VlsE-C6 peptide ELISA. Peptides from three *Borrelia* strains (IP90, PT7, B31) were tested. Samples are grouped according to their reactivity in the commercial immunoblot (negative to highly positive: VlsE^−^, VlsE^+^, VlsE^++^, VlsE^+++^). Values from the in-house ELISA are plotted on the y-axis. ELISA values were normalized to two negative control samples (Ctrl) included on each plate. **(B)** Labeling of memory (CD27^+^IgD^−^) and naïve (CD27^−^IgD^+^) B cells from seropositive and seronegative donors with VlsE-C6-Neutravidin tetramers (52). Background staining with biotin tetramers only was subtracted to give the values shown. Reactivities of samples of the different groups were compared by One-way Analysis of Variance test followed by Tukey's Multiple Comparison test. Results are from five independent experiments including data from nine seropositive and five seronegative donors. Each datapoint represents the percent reactive cells with individual peptides. For an initial assessment, three seropositive and three seronegative donors were only screened with the IP90 peptide (Supplementary Table 1). Therefore, there are 3x1(IP90) + 6x3(IP90, PT7, B31) = 21 datapoints for seropositive donors and 3x1(IP90) + 2x3(IP90, PT7, B31) = 9 datapoints for seronegative donors. **(C)** Tetramers containing the individual peptides were pooled and acute Lyme disease patients' samples stained with otherwise the same protocol. Only part of the healthy controls (*n* = 6, average age: 52, 33% female) and all of the acute patients (*n* = 11, average age: 55, 82% female) were sampled at different timepoints over 1 month period shown as T0, T1, and T2. Samples from single timepoints (T0 for both acute and healthy controls) were compared using a One-tailed Mann Whitney test. Two-tailed unpaired *t*-test showed no difference in age between both groups. **(D)** The percentage of CD27+ and CD27+IgD- memory B cells reacting with VlsE-C6 peptide tetramers of the acute patients shown in panel 2C, presented by time since onset of symptoms. Spearman's Rank Correlation Hypothesis Testing was done manually in Excel. *p = 0.01–0.05, ***p = 0.0001–0.001.

*Borrelia*-reactive memory B cells were directly labeled and sorted using a peptide tetramer staining approach (52) with immunodominant VlsE-C6 epitopes (51) of *Borrelia* that infect humans most frequently in Europe, namely *B. burgdorferi, B. garinii*, and *B. afzelii*. Ninety-five percent of the donors strongly positive for VlsE in the commercial IgG immunoblot were also seropositive for at least one of the three peptides in our in-house ELISA (Figure 2A). The peptide staining approach (for gating strategy see Supplementary Figure 3) was further validated by confirming staining of switched memory B cells (CD27^+^IgD^−^) but not naïve B cells (CD27^−^IgD^+^) in seropositive but not in seronegative donors (Figure 2B). Memory B cells of the seropositive donors were much more reactive (>0.15% stained cells) with the peptide tetramers (56%; 5/9 donors tested) as compared to seronegatives (20%; 1/5 donors tested) (Figure 2B). The staining pattern observed for acute Lyme disease patients (Figure 2C) was similar to the one of seropositive donors (Figure 2B). Also, in acute Borreliosis patients, only a subset of samples showed a detectable increase in peptide tetramer staining (Figure 2C). Interestingly there was a correlation between time of onset of symptoms and the number of tetramer reactive memory B cells (Figure 2D).

### VDJ Heavy Chain Sequences From Sorted VlsE-C6 Reactive B Cells

From 10 acute donors and 4 healthy seropositive or seronegative individuals, RNA of BCR heavy chain transcripts of 628 peptide reactive single B cells and 362 control cells (including peptide negative, NeutrAvidin positive and randomly sorted B cells) were sequenced (for a list of all single cell sequences obtained see Supplementary Materials). There was no difference in CDR3 length distribution or V-gene usage between randomly sorted and the peptide reactive memory B cell subsets. Similar sequences were grouped into clusters using different thresholds of similarity of CDR3aa sequences on either the full V-J gene sequence (100–35% in 5% intervals, 25, 15 and 0% CDR3 aa similarities were tested) (63) or the CDR3 only (92, 83, 80, 75, 67, 50% CDR3 aa similarities were tested) (44). Both approaches showed that unique sequences have been isolated from each donor and that at higher similarity thresholds, peptide and control sequences from individual donors rarely grouped with those of other donors. At the 65% CDR3aa identity threshold 6% (34/616) of sequences from peptide positive cells clustered with 1 to 7 other sequences from peptide-positive cells (Supplementary Figure 4). Only 1% (4/353) of the control cells clustered with another control sequence (Supplementary Figure 4). When analyzing mixed clustering between peptide positive and control cells, sequences of both groups intermixed to a similar extent. For peptide positive 0.97% (6/616), and for controls 0.85% (3/353) of the sequences clustered with at least one sequence from the other group (Supplementary Figure 4). Interestingly, sequences from clusters of seropositive donors were generally more distant from the germline and from each other than sequences (predominantly of the IgM isotype) from the acute patients (Supplementary Figure 4, refer to Supplementary Figure 3 for gating strategies).

### VDJ Heavy Chain Sequences of Stimulated B Cells Reacting With VlsE-C6 Peptide

We further investigated the B cell receptor of B cells showing antibody reactivity toward VlsE-C6 after *in vitro* stimulation (57) at the single cell level. Supernatants of 3840 stimulated single B cells from four acute Borreliosis donors were screened for antibody reactivity against the three *Borrelia* VlsE-C6 peptides as well as for antibodies against other common antigens (including Tetanus toxoid, Cytomegalovirus, Varicella Zoster virus, Measles virus, Rubella, Mumps, Epstein-Barr-Virus, and *Toxoplasma gondii* antigens) as controls (Supplementary Figure 5). With this approach, another 8 VlsE-C6 reactive sequences were identified (Table 1), one of which occurred in two different wells from the same donor and one of which reacted with both *Borrelia* and Measles virus (CDR3-IMGT: ARADDSPSYYVNAFDL) (Table 1). On average 0.07% of wells (Supplementary Figure 5D) reacted with at least one of the three VlsE-C6 peptides [Lyme8: 0.142% (IP90, PT7, B31); Lyme4: 0.071% (IP90), 0.047% (PT7, B31); Lyme6: 0.032% (IP90, PT7), 0.095% (B31); Lyme3: 0.028% (IP90, B31), 0.057% (PT7)].

**Table 1:** Overview of sequences obtained from VlsE-C6 reactive single B cells with *in vitro* single cell stimulation.

**CDR3aa**	**Stimulated sample**	**# Sequences**	**Related clone HTS**	**V-gene**	**J-gene**
ARVVCTGGT	Lyme8 (T1)	1	No	IGHV4-34	IGHJ5
ARGTRDGQ	Lyme8 (T1)	1	No	IGHV3-33	IGHJ1
ARGTRDGQNPEFDY	Lyme8 (T1)	2	Lyme8	IGHV3-33	IGHJ4
ARADDSPSYYVNAFDL	Lyme4 (T1)	1	No	IGHV5-51	IGHJ3
AILWGGLGVVTPDYYYFEMDV	Lyme3 (T1 & T2)	1	No	IGHV1-69	IGHJ6
TRNEIQLDD	Lyme6 (T0 & T1 & T2)	1	No	IGHV3-49	IGHJ4
VRNHTVYVGGNCNSNPGVFDI	Lyme6 (T0 & T1 & T2)	1	No	IGHV3-48	IGHJ3
ARSYEYYYYYAMDV	Lyme6 (T0 & T1 & T2)	1	Control (1 sequence)	IGHV3-7	IGHJ6

### B Cell Receptor Clusters Overlap Between Acute Borreliosis Patients and Controls

**Figure 3:**
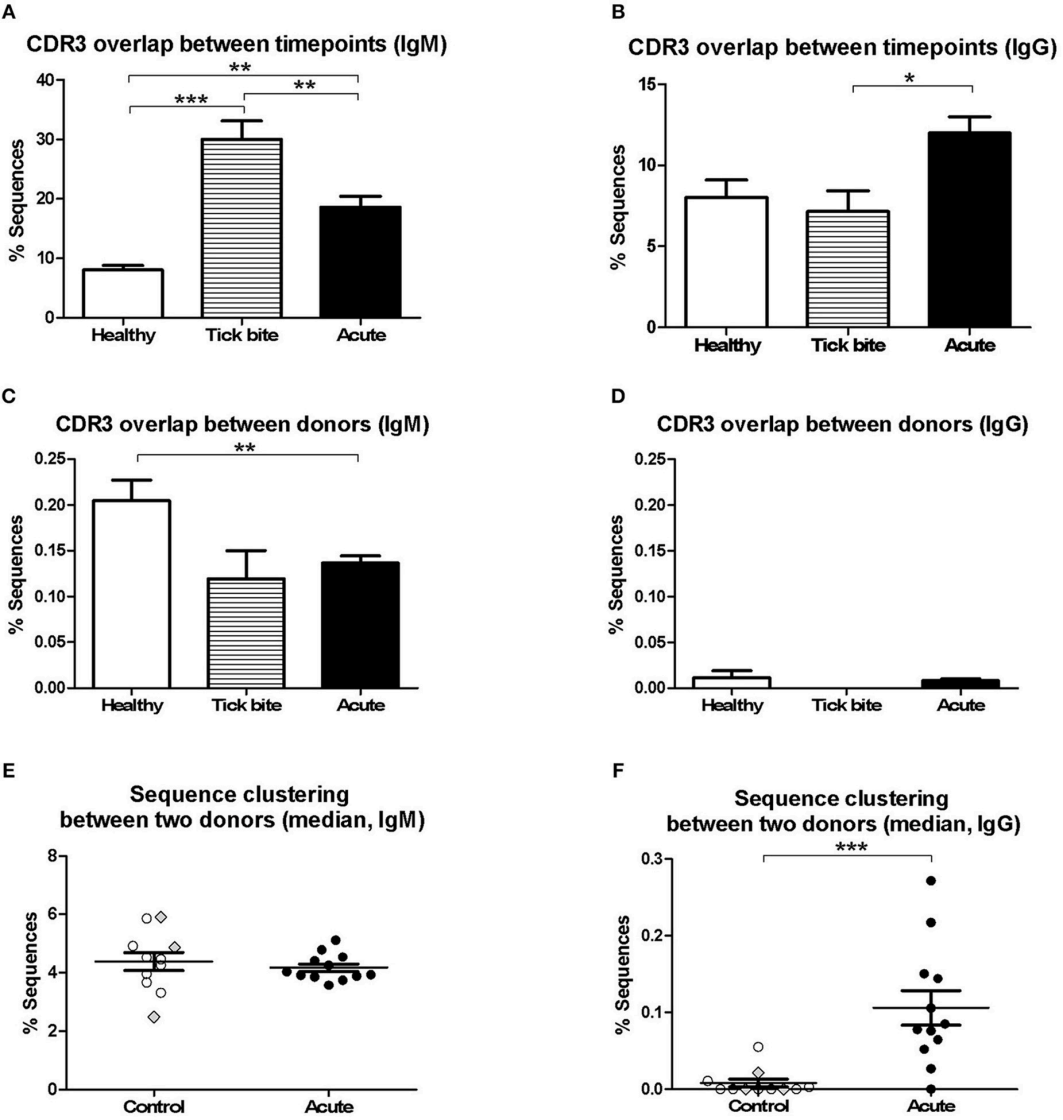
Analysis of pairwise BCR repertoire sequence overlap or clustering between samples with HTS. CDR3aa sequence overlap was determined between samples from different timepoints **(A,B)** and donors **(C,D)**. The overlap between different timepoints (T0-T1, T1-T2, T0-T2) were calculated for each sample and the mean with SEM from all values obtained for each group is represented. Groups were compared with One-way Analysis of Variance test followed by Tukey's Multiple Comparison test. **(E,F)** Sequences were clustered based on whether they contain the same V and J genes as well as similar CDR3aa sequences, allowing one in six mismatches (83% CDR3aa similarity) using the python script from Galson et al. (44, 48, 68). Comparisons were within-group of 12 acute patients (average age: 56, 75% female) and within-group of 11 controls (average age: 55, 27% female). For each donor the percentage sequence overlap between each sample (i.e., timepoint) from that donor with each individual sample of all other donors of the same group was determined. Depending on the number of available timepoints, this gave 25–69 values for each control donor and 66–96 values for each acute patient. The data points represent the median from all these values for each of the donors. Data points corresponding to the three controls with a recent tick bite are highlighted with gray filled rhombi. A Two-tailed Unpaired *T*-test was used to compare values from the two groups. Two-tailed unpaired *t*-test showed no difference in age between both groups. *p = 0.01–0.05, **p = 0.001–0.01, ***p = 0.0001–0.001.

Next, we investigated changes in the bulk B cell repertoire induced by *Borrelia* and the tick bite. We found that 97 ± 1% of CDR3aa sequences (100% sequence identity) overlapped between IgG sequencing replicates, 74 ± 24% overlapped between IgG library replicates and biological replicates overlapped by 32 ± 11% for IgG and 17 ± 10% for IgM. After data processing and consensus sequence building with an in-house pipeline (59), an average of 24012 ± 39051 (median: 9470; min: 594, max: 218302) productive sequences were obtained for IgG samples and 83914 ± 65861 (median: 66878; min: 1727; max: 231257) for IgM. The CDR3aa sequence overlap between the different timepoints of the same individuals was similar to the overlap between biological replicates (on average 8–30% ranging from 0.31 to 59% for IgM and 6–12% ranging from 0 to 35% for IgG) (Figures 3A,B). Interestingly, the IgM overlap was two times higher for the acute patients and three times higher for the tick bite patients on average (Figure 3A). Also for IgG, the average sequence overlap between the different timepoints of acute disease donors was higher than in the case of tick bite and healthy control donors (Figure 3B). As expected (68, 72), interdonor overlap was very small (Figures 3C,D), indicating that each donor has a fairly unique B cell receptor repertoire. IgM CDR3aa sequence overlap was ~1.5 times more frequent in healthy individuals than in the other groups (Figure 3C) and there was essentially no overlap between IgG samples from different donors (Figure 3D).

Because of the uniqueness of the BCR repertoire sequences of each donor, within-group inter-individual sample overlap was also assessed using a less stringent approach Galson et al. (44, 48, 68). Repertoire sequences of the same V-J combination were clustered allowing one mismatch for every six CDR3 amino acids (i.e., 83% CDR3 identity). The intragroup overlap of each pair of samples (all timepoints confounded) of the different donors (606 pairs for the control group and 1120 pairs for the acute group) was calculated. Depending on the number of timepoints available, there were 25 to 69 values for each donor of the control group and 66 to 96 values for each acute donor. For each individual (11 controls and 12 acute), the median of these values was calculated and groups were compared (Figures 3E,F). Interestingly, a significant difference (mean ± SE of the mean of acute: 0.1061 ± 0.02263 and controls: 0.008202 ± 0.005127, *p* = 0.0006, Two-tailed Unpaired *T*-test) between both groups was observed for IgG but not for IgM.

### Comparison of Single Cell Sequences With HTS Sequences

**Figure 4:**
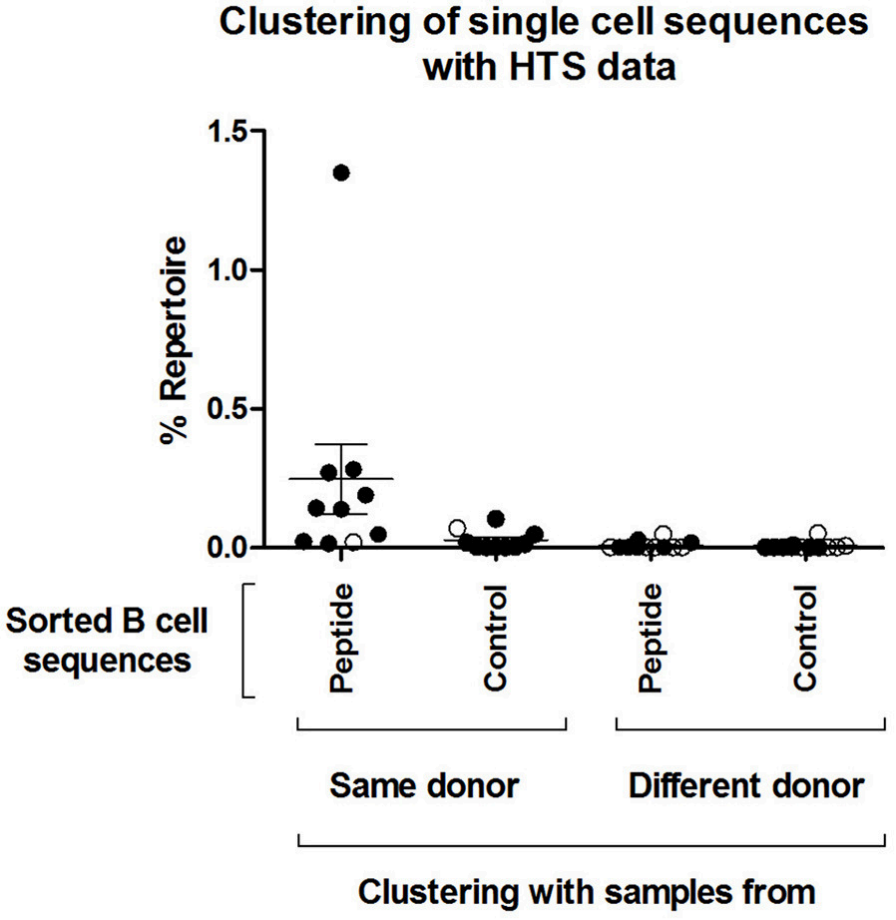
Clustering of single cell sequences with deep sequencing data. Change-O (67) was used for clonal grouping of pooled HTS sequences (referred here to as “Repertoire,” including pooled IgM and IgG sequences from all sequenced timepoints) of each donor with the sequences from the isolated single cells. A clonal grouping threshold was determined with SHazaM R package (67) using the HTS data of each individual donor. This package uses distance to nearest neighbor analysis to determine the threshold. This threshold was then used to cluster the dataset containing both the HTS and all single cell sequences. The percentage of the repertoire (IgM and IgG of all three timepoints confounded) that clustered by this method to all the isolated single cell sequences from the different groups (“Peptide,” “Control”) is shown. The percentage repertoire that is related to the isolated single cells is shown. Values were separated according to whether the sequences were found in the same donor in which they were detected or in a different donor. Open circles: Control samples. Black circles: acute patient samples.

**Figure 5:**
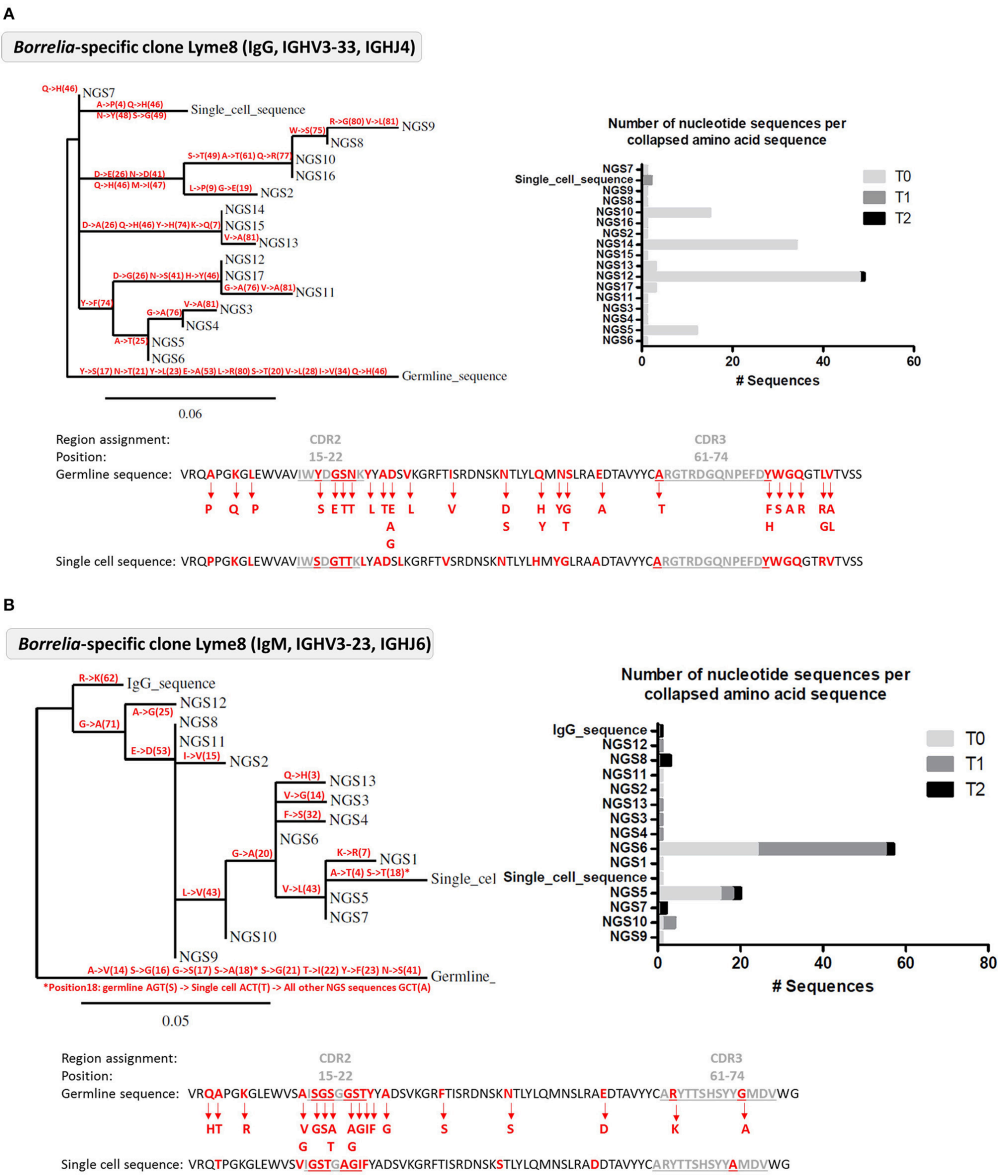
Analysis of *Borrelia* VlsE-C6 reactive B cell clones detected in the deep sequencing data from Lyme 8 patient. **(A)** Clone related to the single cell isolated by *in vitro* single cell stimulation. **(B)** Clone related to single cell isolated with tetramer staining approach. **(A,B)** Left: Phylogenetic tree highlighting the mutations (with the positions of the mutations in brackets) occurring at each branch of the tree. Right: Number of nucleotide sequences that made up each amino acid sequence represented in the phylogenetic tree as well as the sampling timepoint at which they were detected. Sequences have the same order than in the phylogenetic tree. Bottom: The VDJ sequence of the germline and the isolated single cell sequences are shown. Amino acid substitutions from the clone are highlighted in red.

Next, we tested whether the VlsE-C6 reactive single cell sequences isolated with the tetramer approach can be retrieved in the deep sequencing data. For this purpose, HTS from all collected timepoints (T0: ~diagnosis, T1: 1 week after T0, T2: 4 weeks after T0) and the different isotypes were combined with sequences from isolated single cells and the Change-O pipeline was used for clonal grouping (67). With this approach, the vast majority (81%, 130/160) of the single cell sequences that were found in the HTS data, grouped with sequences of the same donor (Peptide reactive sequences with same donor: 96, Control sequences with same donor: 34) while only a few grouped with sequences from another donor (Peptide reactive sequences with different donor: 15, Control sequences with different donor: 15). In acute donors, up to 1.5% of all pooled IgM and IgG sequences grouped with a peptide reactive single cell sequence of the same donor and <0.1% with a peptide reactive single cell sequence of a different donor (Figure 4). This further indicates that the BCR repertoire of an individual is very “private.” The clones related to sequences from the VlsE-C6 enriched B cells were generally not among the top clusters of the acute patients. A similar analysis was done with the sequences of VlsE-C6 reactive stimulated B cells. For two of the eight VlsE-C6 IgG sequences obtained with the stimulation approach, related sequences were found in the HTS data (Table 1). A single naïve IgM sequence with a CDR3 similar to the VlsE-C6 reactive ARSYEYYYYYAMDV was found in a control donor. Interestingly, the VlsE-C6 reactive CDR3 ARGTRDGQNPEFDY, which could be isolated from two different VlsE-C6 reactive wells after simulation, could also be found in a large cluster of 123 sequences (0.0292% total repertoire) within the deep sequencing data of the same donor (Lyme8) (Figure 5A). All sequences of that cluster were of IgG isotype. The length of the phylogenetic tree trunk, suggests that this clone may have started from an already mutated B cell. Most single cell sequences (including those reacting with the control antigens) obtained by *in vitro* single cell stimulation (82%, 32/39) were not found back in the HTS data, probably because the latter is incomplete and skewed toward cells containing large amounts of immunoglobulin transcripts.

### Clonal Analysis of the B Cell Repertoire HTS Data

**Figure 6:**
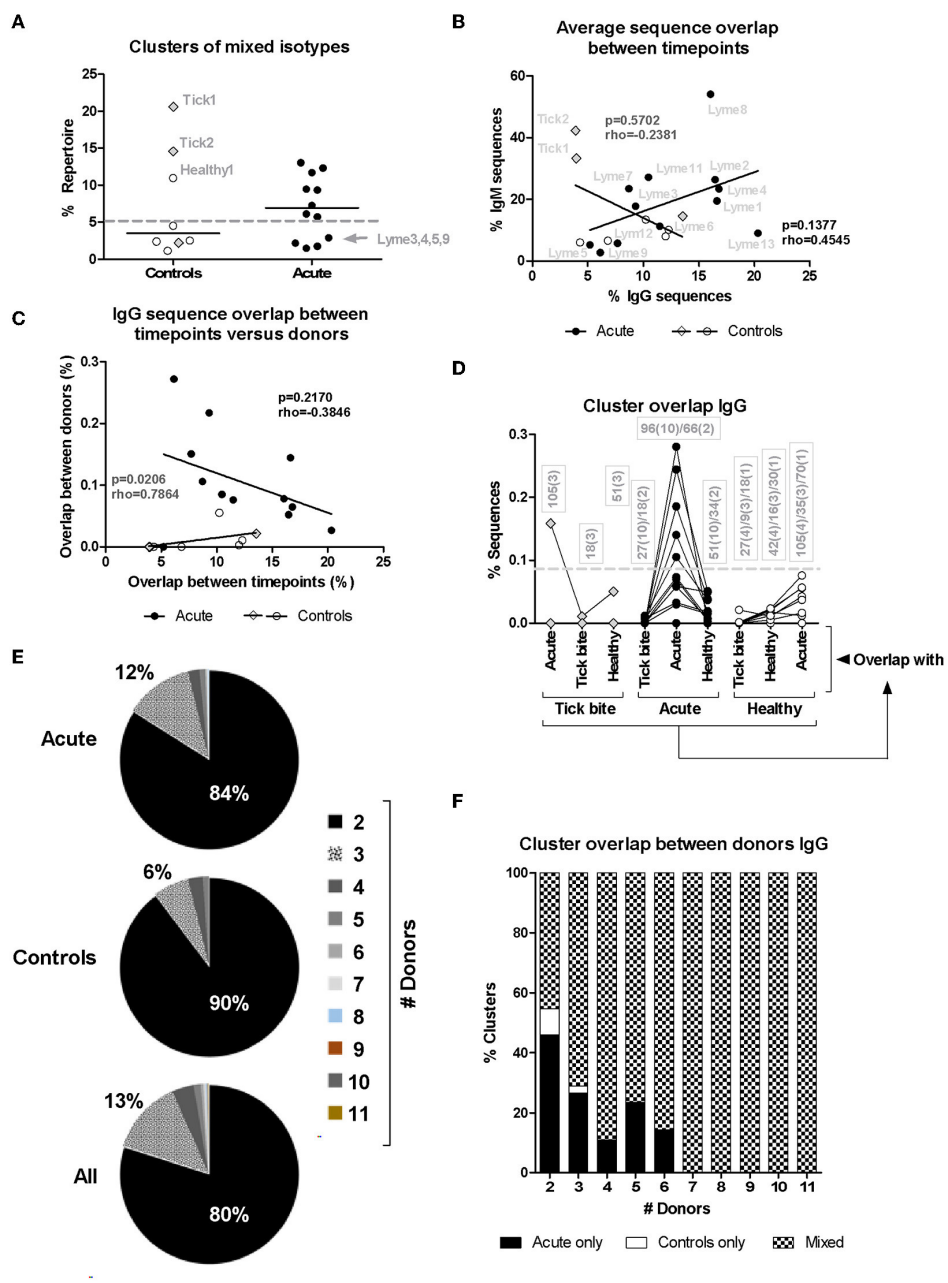
Analysis of HTS sequence clustering. **(A)** Mixed clones obtained using Change-O (67) that contained sequences of both IgM and IgG isotypes were selected and the percentage of the total repertoire (all samples from the same donor pooled) they represent is shown. **(B)** Correlation between average IgM and IgG CDR3aa sequence overlap between timepoints for the different patients (closed symbols) and controls (open symbols). **(C)** Correlation between IgG CDR3aa sequence overlap between timepoints (average) and clustering between donors (median) (44, 48, 68) is represented. Spearman's Rank Correlation Hypothesis Testing was done manually in Excel. **(D)** Sequence clustering between all donors was determined using the python script from Galson et al. (44, 48, 68). Median values were calculated as before (Figure 4) but here the sequence overlap between samples belonging to different groups was also assessed. Lines connect values of the same donor. The boxes show the number of sequence overlap values calculated and used to generate the median values presented. The brackets give the number of donors for which this number of sequence overlap values was used to calculate the median value shown. Dashed line: 5/12 (42%) acute patients but only 1/11 (9%) controls showed median values above this threshold. **(E,F)** Distribution of overlapping clusters among donors.

The higher CDR3aa sequence overlap between samples from different timepoints observed for acute patient's IgM and IgG repertoires (Figures 3A,B) suggested ongoing B cell immune responses at both of these levels. Since B cell phenotyping showed rather heterogeneous B cell responses between donors (Figure 1), we used bulk BCR sequencing to investigate whether there may be a correlation between the extent of IgM and IgG responses or whether individual donors react rather at one or the other of these two levels. When HTS IgM and IgG sequences from a same donor were pooled, only 20% (1/5) of healthy donors but 67% (8/12) of acute patients had high numbers of sequences (more than 5% of total repertoire sequences) within clusters containing both IgM and IgG isotypes (Figure 6A), indicative of an immune response with isotype class switching.

There was no significant correlation (rho = 0.4545, *p* = 0.1377, Spearman's Rank Correlation Hypothesis Testing) in the patient group when plotting IgM vs. IgG sequence overlap between samples of the same donor but from different timepoints (T0: ~diagnosis, T1: 1 week after T0, T2: 4 weeks after T0, Figure 6B). The four donors with modulated VlsE-C6 IgG titers (Lyme8 & Lyme2↑, Lyme 4 and Lyme13↓, Lyme1↑↓) showed, however, the highest IgG CDR3aa sequence overlap between the different timepoints (Figure 6B). There was also no correlation (rho = −0.2381, *p* = 0.5702, Spearman's Rank Correlation Hypothesis Testing) in the controls (Figure 6B). Two tick bite donors showed a high CDR3aa sequence overlap between timepoints at the IgM but not at the IgG level.

The results in Figures 3A,B showed the presence of a larger number of expanded B cell clones in acute patients than in controls. As there was also a higher intragroup IgG sequence cluster overlap between acute donors (Figure 3F), we investigated whether there was a correlation between clonal expansions (Figure 3B) and overlap with other acute patients (Figure 3F). If different donors indeed produced similar clones in response to infection, one could expect for donors with the strongest response also a higher chance to produce sequences overlapping with other donors. After having defined for each donor the average overlap between samples from the three different timepoints (Figure 3B) and calculated the median of the overlaps between within-group pairs of individuals for each donor (Figure 3F), we correlated both parameters. There was no correlation between these two parameters (Figure 6C), but when the one outlier patient (Lyme5) was excluded, there was a strong negative correlation (rho = −0.8000, *p* = 0.0031, Spearman's Rank Correlation Hypothesis Testing) for the rest of the Borreliosis patients. Lyme5 showed no sequence overlap with any of other donors, probably because of the low sequence numbers recovered from this donor.

In order to exclude that a higher sequence overlap may be due to a larger number of repertoire samples being compared intragroup among acute patients than intragroup among controls (Figure 3F), we clustered IgG sequences from samples of all donors of both groups and assessed (as in Figure 3F) for each donor the degree of overlap with each donor from the same but also other groups. Overlap values obtained for individual samples of all three timepoints (T0: ~diagnosis, T1: 1 week after T0, T2: 4 weeks after T0) were used to calculate median overlap values for each donor with each group (“Acute,” “Healthy,” “Tick bite”). Acute patients showed the highest overlap only with members of their own group (except for one tick bite donor) and samples from other groups did not reach this level even if comparing to a larger number of repertoires (Figure 6D). Most clusters overlapped only between two individuals (Figure 6E) and most clusters overlapping between more than 2 acute patients were also present in healthy donors (Figure 6F). When focusing on the top 50 clusters of each sample, 58 of 1700 clusters (34 samples; 3.4%) overlapped with sequences from another acute donor. The corresponding sequences from the other repertoires did normally not belong to the top 50 clusters. Two clusters particularly stood out: The first cluster [CDR3aa consensus: X-K(R)-W-R-X-X-Q-S-E-X-D(E)-X; IGHV3-7; IGHJ4] was found in 5 different donors [Top50: Lyme7 (T2), Lyme12 (T1); Top200: Lyme4 (T2), Lyme11 (T0); low level: Lyme1 (T0,T1,T2), Lyme4 (T1), Lyme12 (T0,T2), Lyme7 (T0)]. The second one (CDR3aa consensus: A-K-X-X-X-X-X-C-S-X-X-X-C-Y-X-F-D-X, IGHV3-23; IGHJ4) was found to overlap even between 6 different donors [Top50: Lyme3 (T2), Lyme9 (T2); Top200: Lyme9 (T0), Lyme 8 (T0), Lyme12 (T0); low level: Lyme2 (T1); Lyme7 (T0, T1)]. Both of these clusters were not found in any of the control samples.

## Discussion

In the present study, erythema migrans was used as a common pathognomonic denominator and virtually all donors (12/15) had antibodies against VlsE as determined by immunoblot or reacted with VlsE-C6 peptides by ELISA (Supplementary Table 1). Nevertheless, patients differed by time of infection until diagnosis and disease progression. At least in a subgroup of the patients, activated B cell subsets including plasmablasts, CD23 expressing naïve B cells and IgG^+^ memory B cells were increased (Figure 1), but there was no clear expansion of a defined B cell subset common to all acute patients. Overall this is in line with previous observations (10, 24–28), that *Borrelia* elicit a heterogeneous B cell response typically detected only in a subset of patients.

In order to reduce antigen complexity, we focused on the VlsE-C6 epitope, which is conserved among different *Borrelia* species (73). This antigen is expressed throughout the course of the infection (18) and VlsE is commonly used as antigen in diagnostic tests. Our three peptides (51) cover most reactivities, since 95% of donors with a strong VlsE-reactivity in a commercial immunoblot also showed reactivity in our in-house peptide ELISA (Figure 2A). VlsE-C6 reactive B cells were obtained by either peptide tetramer staining or by *in vitro* single cell stimulation. After *in vitro* single cell stimulation of CD20^+^CD27^+^IgM^−^IgA^−^IgD^−^ memory B cells, levels of VlsE-C6-reactive B cells were similar or lower compared to control antigens of vaccine preventable pathogens (Supplementary Figure 5D). On average 0.07% of stimulated cells reacted with a VlsE-C6 peptide (Supplementary Figure 5D). Thus, VlsE-C6 reactive B cell clones are rare events in the patient's repertoires, and the majority of isolated CDR3s cannot be retrieved in the HTS data of the same or a different donor (Table 1). Only in a single donor (Lyme8) which showed a strong increase in VlsE-C6 IgG antibody titer increase between the sampled timepoints in the peptide ELISA (Supplementary Figure 6) the same CDR3aa was found. The related HTS clone identified in Lyme8 (Figure 5A) was the fourth largest IgG clone (123 sequences) in this patient's IgG repertoire. Also, after tetramer staining we sorted a single cell related to a large (94 × IgM, 1xIgG sequences) cluster of clonally related B cells from this donor (Figure 5B). In other patients, VlsE-C6-tetramer-reactive sequences could be identified sporadically among the top clones of an isotype specific repertoire. Thus, both single cell approaches confirmed a low and heterogeneous response of private CDR3 sequences toward *Borrelia*. There was a measurable increase in tetramer reactive cells with time after onset of symptoms (Figure 2D), reflecting the expected expanding cellular response after infection.

The above analysis indicates, that when only a limited number of B cell receptors of single VlsE-C6 reactive B cells obtained either by flow cytometry or by *in vitro* clonal expansion are sequenced, different individuals generate B cells with different CDR3s even toward the same epitope (Supplementary Figure 4, Table 1). Clustering analysis of the single cell sequences with the deep sequencing data showed, that the majority (81%) of those that were related to an HTS clone clustered with sequences from the same donor (Figure 4, Table 1). The 17–32% CDR3aa sequence overlap observed between biological replicates is comparable to other next generation sequencing analysis of patient's B cell repertoires (50, 68) and indicates that our HTS approach covers a significant part of the expanded repertoire. Thus, our HTS cluster analysis (Figure 4, Table 1) further indicates that the clones from which the VlsE-C6 reactive B cell repertoires emerge are rather unique or private for each patient. To further investigate this, we analyzed sharing of identical CDR3aa sequences between donors by direct comparison of HTS data from the same (Figures 3A,B) and different (Figures 3C,D) patients and controls. A CDR3aa overlap of ~10–30% was observed between samples from different timepoints of the same donors, but the overlap between donors on average did not exceed 0.2%. When calculating CDR3aa sequence overlap between samples collected at different timepoints from the same donor, a significantly higher sequence overlap between acute patient samples from different timepoints as compared to controls was observed (Figures 3A,B). This indicates that these CDR3s are part of an ongoing clonal expansion. Sequence clustering (44, 48, 74) allows to group B cell repertoire HTS sequences into clones using less stringent similarity criteria (i.e., 83% CDR3aa similarity and same V-J gene combinations instead of identical CDR3aa sequences). This revealed a difference in sequence overlap between the groups (Figures 3F, 6D), at least at the IgG level. Since a higher cluster overlap between individual samples occurred only rather sporadically, we excluded that the difference observed in Figure 3F was due to a larger number of samples being compared for acute patients than for controls. First using median instead of mean for overlapping values buffered the impact of outliers. Furthermore, we compared cluster overlap among members across the different groups (Figure 6D). This allowed to compare overlap between a larger number of samples, also for the control group. With this approach, we found that among the controls, only one tick bite donor reached the level of cluster overlap observed between acute patients. This high overlap was observed when comparing its repertoire with the one of acute patients (Figure 6D) and could be explained by an ongoing immune response toward the tick bite.

Both multicolor flow cytometry (Figure 1H) and HTS data (Figures 3A, 6A) showed evidence of an ongoing IgM immune response in at least a subset of the patients. The clustering approach generally showed a much higher interdonor overlap for IgM (Figure 3E) than for IgG (Figure 3F). In the case of IgM, for a cluster to be included into the group of overlapping sequences, it was considered sufficient that only one related naïve B cell sequence was present in the repertoire of the other donor. This may explain why we only saw a difference in sequence overlap between the groups for the IgG repertoire but not for the larger and more diverse IgM repertoire.

The five donors that showed changes in VlsE-C6 IgG titers over the sampling period also showed the highest IgG CDR3aa sequence overlap between the different timepoints (Figure 6B), suggesting that molecular sequencing of CDR3s may support serological findings. There was clearly a negative correlation (Figure 6C), between IgG sequence overlap among different timepoints and sequence clustering between donors. Those results (Figure 6C) seem to be in line with other HTS studies suggesting, that repertoire overlap occurs not at the plasma cell burst on day 7 (the focus of most B cell repertoire studies) but rather at the level of low frequency memory B cells (44). This is also in agreement with the incidental expansion of suboptimal B cell clones that undergo affinity maturation of their B cell receptors in the course of the immune response (75–78).

Furthermore, our data indicate that clones (Figures 6E,F) are only sporadically found to overlap between pairs of acute patients. This mirrors the clustering of antigen-reactive single cell sequences within the HTS data (Figure 4). It remains unclear whether this is due to the low levels of these sequences within the B cell repertoire or whether different donors generate different clones.

We cannot exclude that the sampling or processing of the PBMCs (e.g., heparin) may have had some activating or modulating effect on the BCR, which could explain between-group overlap but not differences of within-group comparisons.

One of the limitations of the present study is the relatively low number of acute patients and the heterogeneity of the patients with respect to timepoints of diagnosis and sampling, disease manifestation, the diversity of the *Borrelia* species in ticks in Luxembourg (49), and the limited statistical power of the sample size.

Our observation that every donor's repertoire is rather unique is in line with previous findings from single cell B cell studies (56, 79–84) as well as from other HTS studies (50, 68, 72, 85–90).

Only very recently, the first study on acute Lyme disease patient B cell repertoire was published by Blum et al. (28). They observed an increased clonal expansion in acute Lyme disease patients as compared to controls, the degree of which could be associated with resolution of symptoms (the higher the plasmablast clonal expansion, the shorter the duration of symptoms). They analyzed patients from the United States, where different major *Borrelia* species cause Lyme disease, and used different sequencing and analysis approaches. Nevertheless, we also report a higher clonal overlap between acute Borreliosis patients as compared to controls. Furthermore, similar to their findings, our flow cytometry data (Figure 1E) indicate, that there may be plasmablast expansions in acute Borreliosis patients but only in a subset of them. Also their data indicate that overlapping clusters are not necessarily common to all repertoires of acute Borreliosis patients and that we will need to focus on B cell subsets of interest, which is also in line with our observation (91).

In conclusion, despite heterogeneous and weak immune responses, we detected a higher CDR3aa sequence overlap between different timepoints for acute patients than in healthy controls. We furthermore observed a higher BCR sequence clustering among IgG samples from acute Borreliosis patients as compared to the control group. Collectively, these data indicate, that HTS of B cell repertoires of acute Borreliosis patients allows to detect ongoing B cell immune responses. We have also identified *Borrelia*-reactive CDR3aa sequences, but these seem to be rather unique for each patient and therefore hardly suitable as biomarkers of an already complex diagnosis.

## Ethics Statement

The study was approved by the competent ethical committee of Luxembourg (Comité National d'Éthique de Recherche, CNER). All donors signed an informed consent sheet. In addition a notification and authorization was submitted and accepted by the Commission nationale pour la protection des données (CNPD) in Luxembourg.

## Author Contributions

JK managed the project, designed and performed experiments, interpreted data and wrote the manuscript. AC designed and performed experiments and provided substantial strategic support. J-PB and WF adapted the HTS data processing pipeline to the barcoding approach. WF performed clustering analysis using the Change-O framework. J-PB developed the ssUID barcoding technique for HTS library preparation. RS setup the deep sequencing platform and performed initial deep sequencing experiments. AD developed B cell repertoire sequencing and data analysis strategies and provided significant strategic support. AR designed the initial project, acquired funding, ethical approvals and supervised the initial part of the study. CM supervised and coordinated the project. All authors contributed to manuscript revision, read and approved the submitted version.

### Conflict of Interest Statement

The authors declare that the research was conducted in the absence of any commercial or financial relationships that could be construed as a potential conflict of interest.

## Abbreviations

BCR: B cell receptor
CDR3: Complementarity determining region
CDR3aa: CDR3 amino acid sequence
FACS: Fluorescence activated cell sorting
HTS: High throughput sequencing
IgG: Immunoglobulin G
IgM: Immunoglobulin M
LIH: Luxembourg Institute of Health
MID: Molecular identifier
PBMCs: Peripheral blood mononuclear cells
QSC: quality score
ssUID: single sided UID
T0/T1/T2: timepoint 0/1/2
UID: Unique molecular identifier
VDJ: Variable (V) Diversity (D) Joining (J) gene segments of immunoglobulin sequence
VlsE: Variable major protein-like sequence expressed
VlsE-C6: peptide representing IR6 of VlsE.
