# Supplementary material for: Detection of a Low Level and Heterogeneous B Cell Immune Response in Peripheral Blood of Acute Borreliosis Patients With High Throughput Sequencing
Source: Front Immunol. 2019 May 16;10:1105. doi: 10.3389/fimmu.2019.01105 (PMC6532064; doi:10.3389/fimmu.2019.01105)
Supplement: Supplementary file 1 [file Data_Sheet_1.PDF]

Supplementary Figure 1

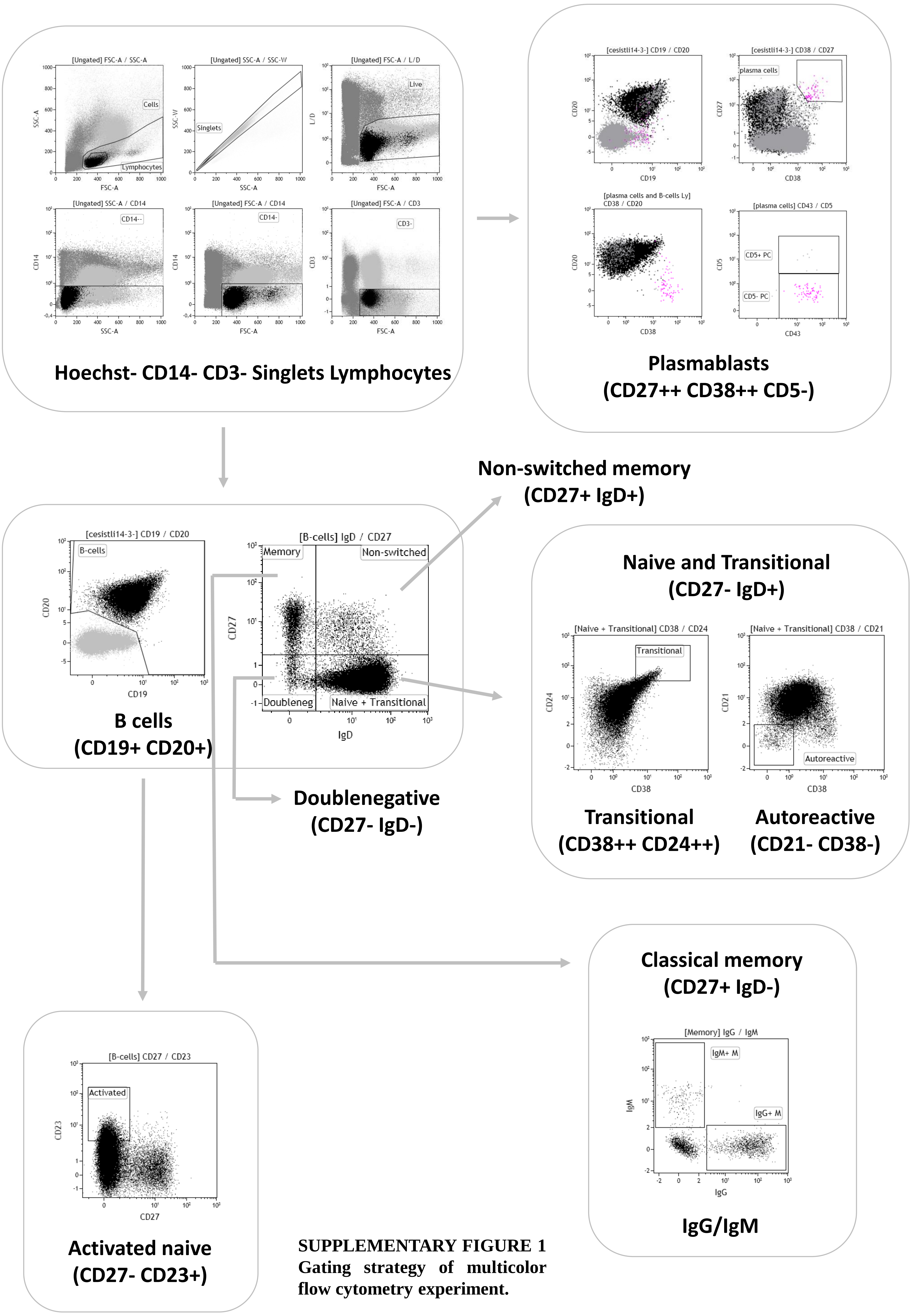

SUPPLEMENTARY FIGURE 1
Gating strategy of multicolor flow cytometry experiment.

Supplementary Figure 2

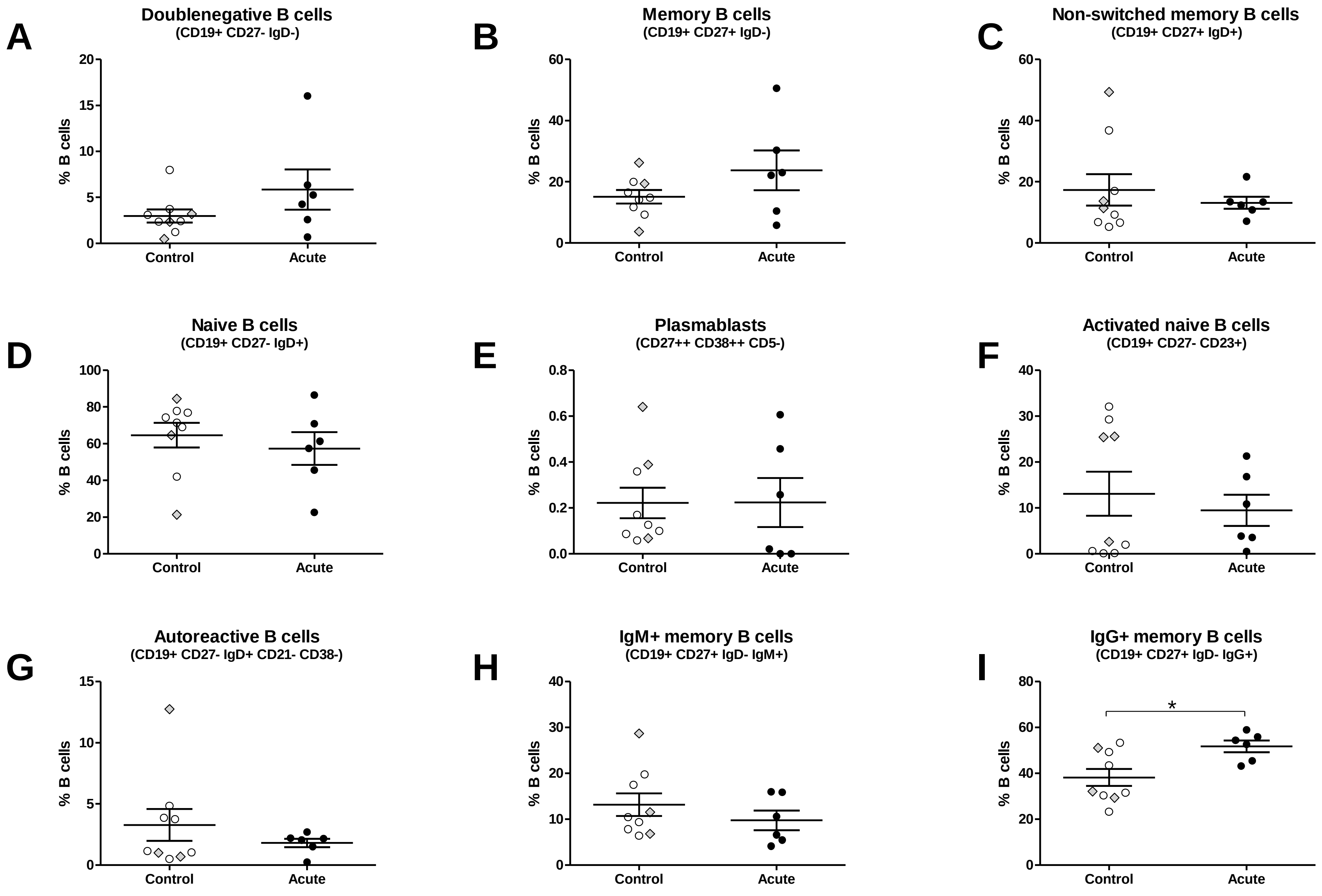

SUPPLEMENTARY FIGURE 2 Details on statistical analysis performed for Figure 1. For the “Comp ctrl” donor the average value from the three experiments was calculated and this donor included into the “Control” group. For the tick bite donors the values obtained with samples from T3 were included into the “Control” group. This is the timepoint that is the furthest away (1 month) from the tick bite, hence closest to “healthy status”. For acute Lyme disease patients, the first timepoint sampled from acute Lyme disease patients was included into the “Acute” group. This is the timepoint closest to diagnosis and start of treatment, hence reflects patients in their acute stage. Control and Acute groups were compared with a Two-tailed Unpaired t test.

Supplementary Figure 3

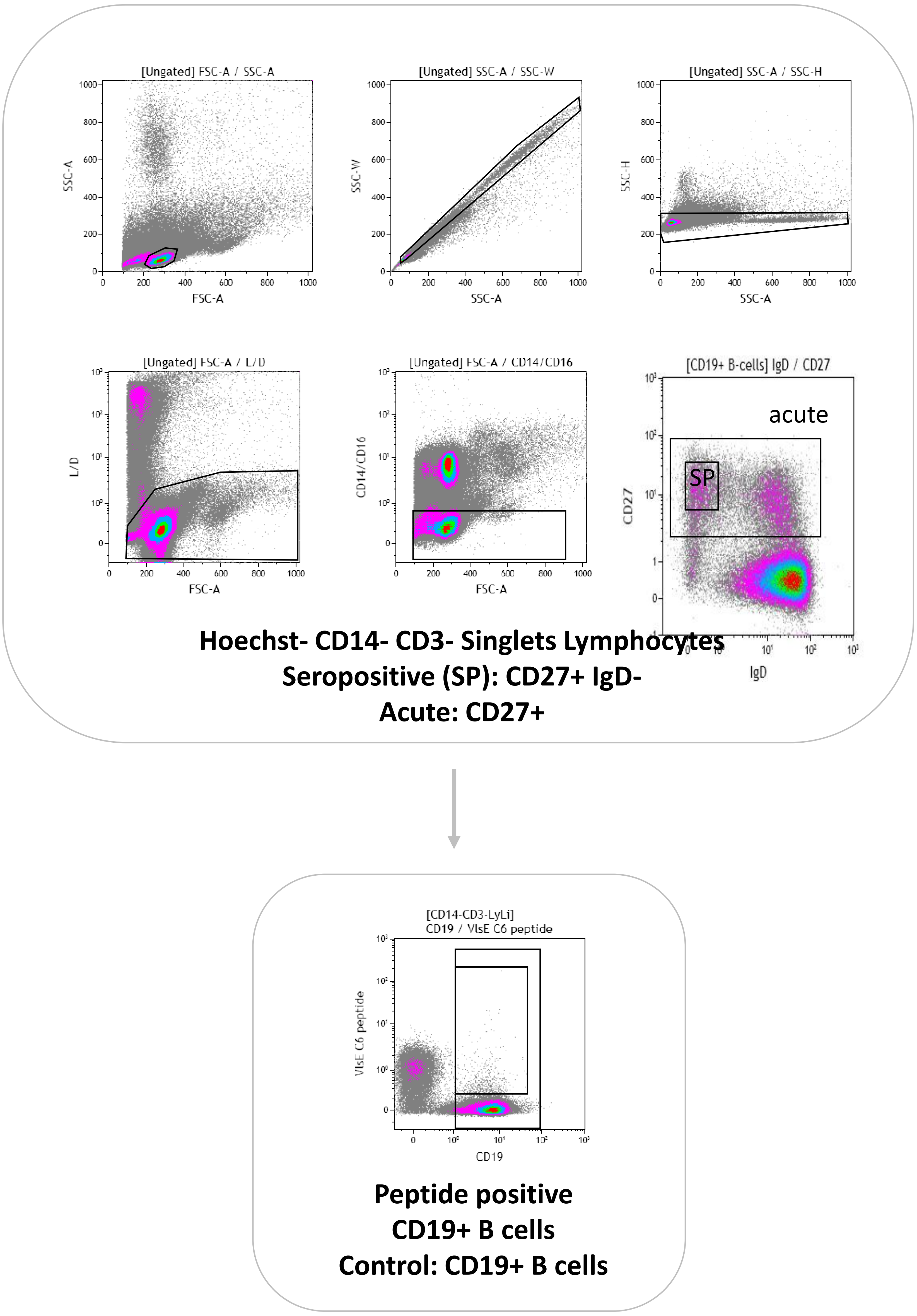

SUPPLEMENTARY FIGURE 3 Representative gating strategy for antigen-specific B cell sorting using VlsE-C6 tetramer staining. For seropositive ("SP") control samples only CD27+IgD- memory B cells that were positive for the peptide were sorted, while for the acute patient samples ("acute"), the gate was enlarged to CD27+ B cells irrespective of IgD expression.

Supplementary Figure 4

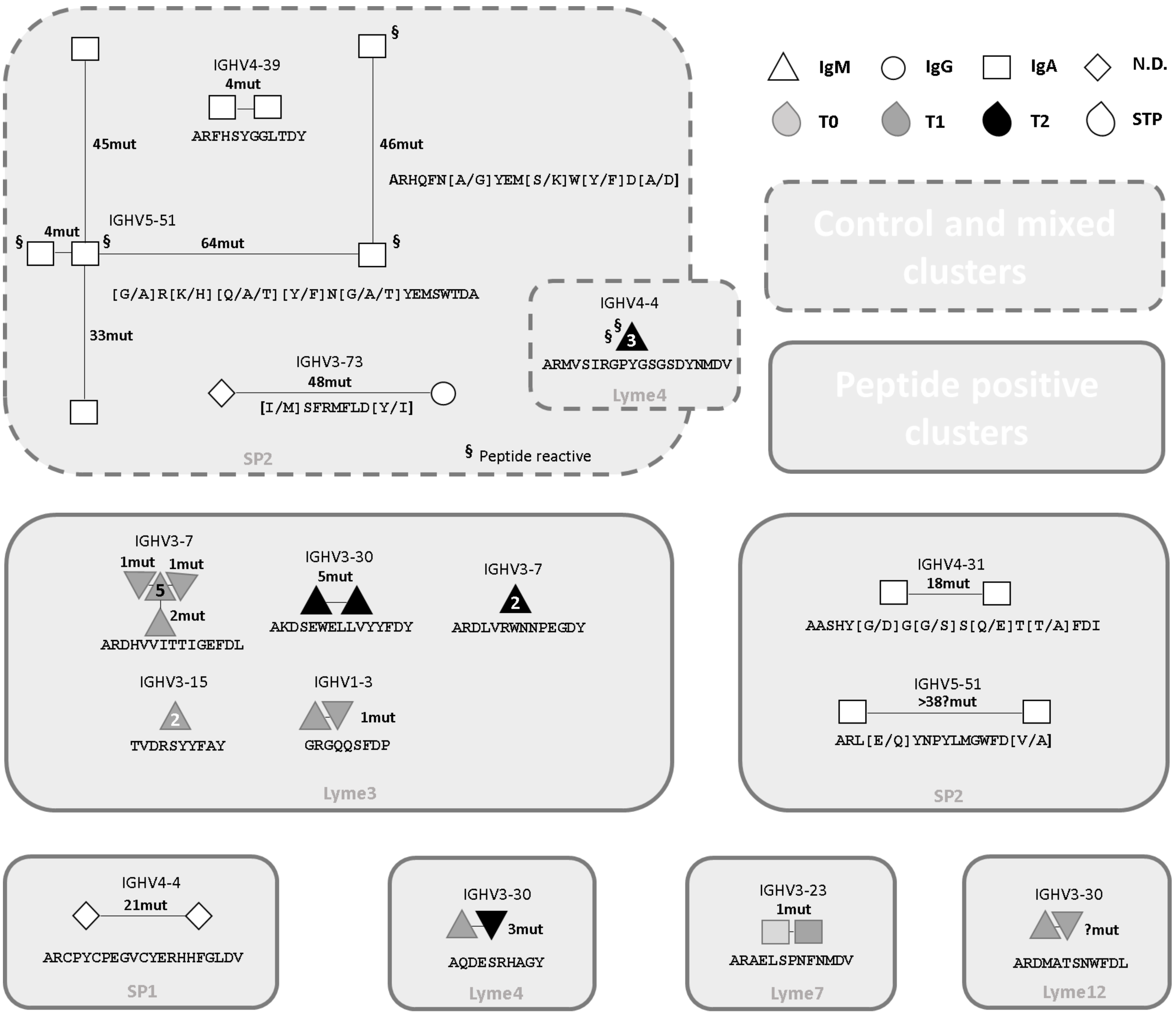

SUPPLEMENTARY FIGURE 4 Representation of single cell sequence clusters. Clusters obtained from single B cell sequences with the BcRep R package (64) at the 65% CDR3aa identity threshold are shown. This figure represents the sequences, the isotype and the timepeoint of sampling (T0, T1, T2), the number of mutations ("distance") separating related sequences (e.g. 3mut) and the germline V-gene (e.g.: IGHV4-39) from which they are derived. The germline V-genes are shown for each cluster. Different timepoints are highlighted in different grey shades. Isotypes are depicted in different shapes. N.D.: isotype not determined because of incomplete sequence, STP: single timepoint measured only, mut: number of mutations separating the linked sequences. The length of each bar is proportional to the number of mutations between the sequences. In mixed clusters, sequences from peptide positive cells are marked with §.

Supplementary Figure 5

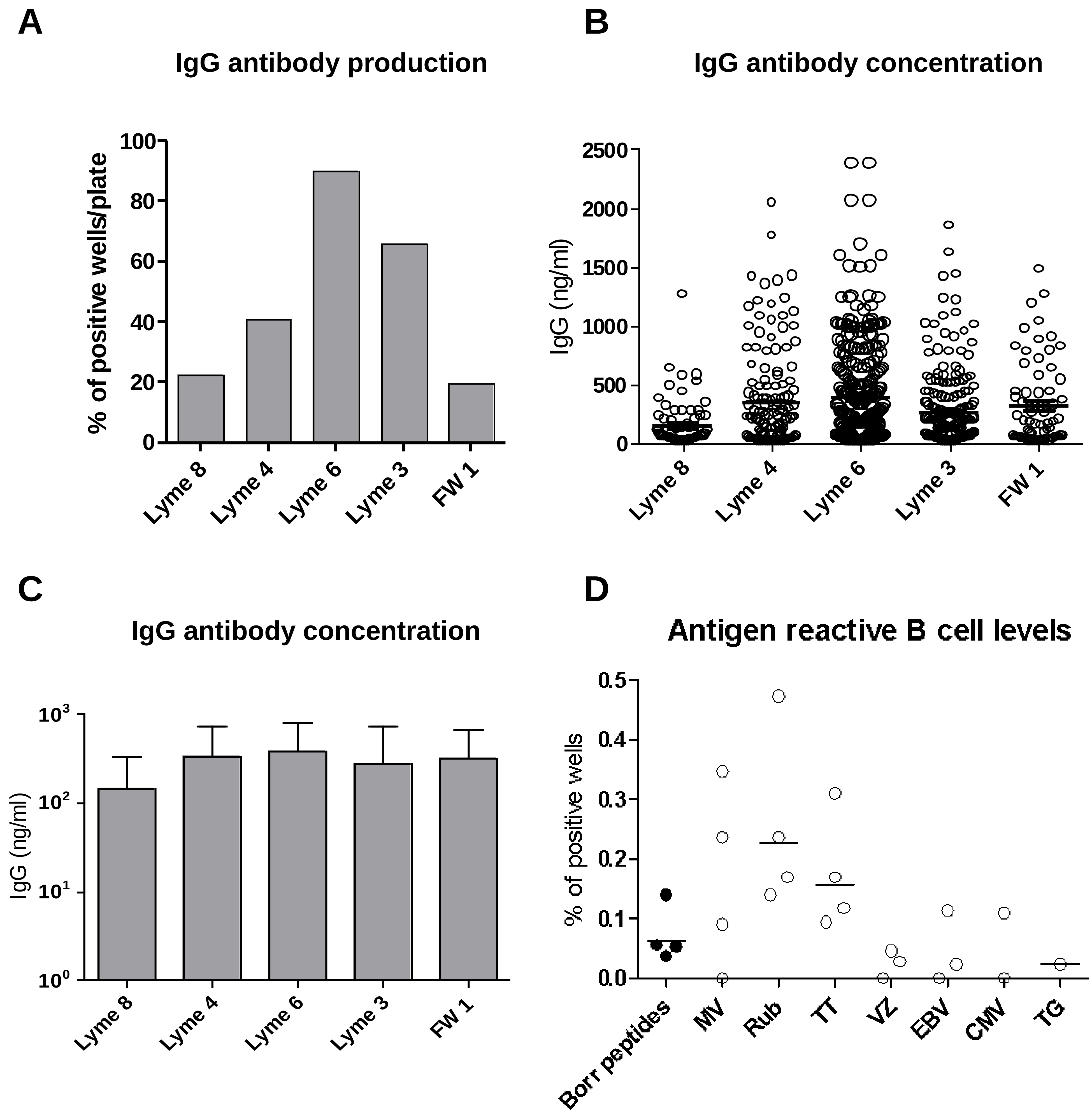

SUPPLEMENTARY FIGURE 5 ***In vitro*** **B cell stimulation.** (A) Total IgG production in individual stimulated wells was assessed for one 384 well plate from each donor using the commercially available and quantitative Human IgG total Ready-SET-Go!(R) ELISA (eBioscience). Represented is the percentage of wells from one plate of each donor that showed IgG production as measured by ELISA. (B) Mean and Standard error of the mean of antibody concentrations from wells positive by total IgG ELISA. Individual values from different wells are highlighted. (C) Mean and standard deviation of data from (B). (D) Percentage wells from single cell stimulation that were positive in ELISAs for the indicated antigens or pathogens. Borr peptides: *Borrelia* VlsE-C6 peptides from either B31, PT7 or IP90 strain; MV: measles virus; Rub: Rubella; TT: Tetanus toxoid; VZ: Varicella-Zooster virus; EBV: Epstein Barr virus; CMV: Cytomegalovirus; TG: Toxoplasma Gondii. Filled black circles: *Borrelia* peptides; Open circles: other antigens or pathogens.

Supplementary Figure 6

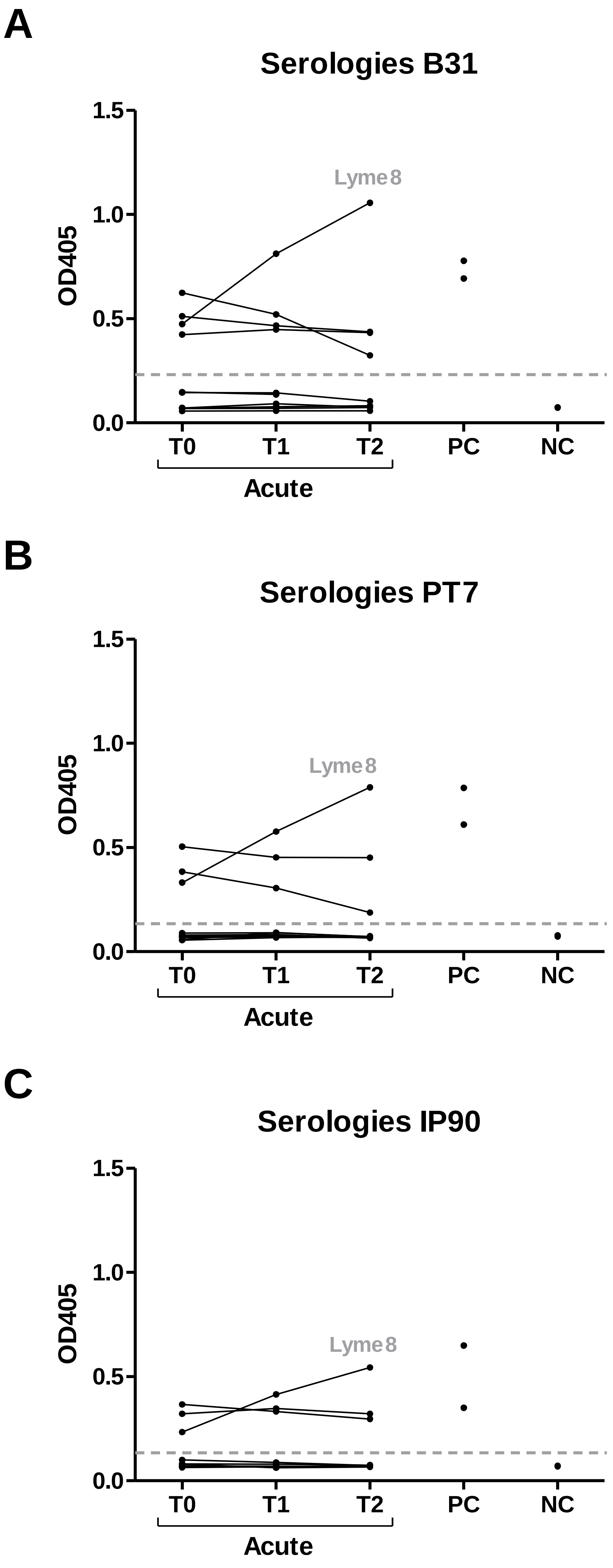

SUPPLEMENTARY FIGURE 6 VlsE-C6 peptide ELISA results obtained for the 10 acute Lyme disease patients from which VlsE-C6 peptide tetramer positive memory B cells were sorted and sequenced. Of these donors only Lyme 8 showed an increase in antibody titers over the sampling period. Two positive control samples (PC) and two negative control samples (NC) were included into each ELISA experiment. Acute: samples from acute Lyme disease patients sampled at the following timepoints: T0: ~diagnosis, T1: 1 week after T0, T2: 4 weeks after T0.

Supplementary Table 1

SUPPLEMENTARY TABLE 1 Detailed overview of acute and control donors analysed in individual experiments. The donors summarized in Tbale 2 are the ones used for the key experiments, meaning multicolour B cell panel and HTS. *For each sorting experiment, seropositive and seronegative controls with similar age were selected from the larger high risk cohort.

Donor	Donor info				Multicolour flow cytometry	VlsE-C6 peptide			HTS	in-vitro single cell stimulation
	Disease manifestation	Two-tier testing (Europe, Genzyme Virotech)	in-house VlsE-C6 ELISA	Age		setup	sorting	test acute		
Lyme1	~2 months Erythema migrans + other symptoms	IgM:?#; IgG:VlsE, (DbpA)*	+/-	50					x	
Lyme2	~2 months Erythema migrans + other symptoms	IgM?§; IgG: VlsE, p39*, DbpA, p58, p83	+	65					x	
Lyme3	~2 months Erythema migrans + other symptoms	IgM: VlsE, IgG: VlsE, (p83)	-	54			x	x	x	x
Lyme4	3 weeks Erythema migrans + other symptoms	IgM: OspC, VlsE; IgG: OspC, VlsE	+ (B31, PT7) - (IP90)	62			x	x	x	x
Lyme5	2-3 months Erythema migrans	Negative	-	50			x	x	x	
Lyme6	2 weeks Erythema migrans	Negative	-	24			x	x	x	x
Lyme7	1 week Erythema migrans + other symptoms	IgM: VlsE, p39; IgG: VlsE	-	43			x	x	x	
Lyme8	4 days Erythema migrans	IgM: (VlsE)*; IgG: VlsE	+	63			x	x	x	x
Lyme9	~3 months Erythema migrans + other symptoms	IgM: OspC, VlsE; IgG: (OspC), VlsE, (p58),(p83)	-	55			x	x	x	
Lyme10	Erythema migrans	IgM: OspC*, (VlsE)*	-	54			x	x		
Lyme11	1 year Erythema migrans + other symptoms	IgM: (OspC); IgG: VlsE, DbpA, (p58)	+	63	x		x	x	x	
Lyme12	2 months Erythema migrans	IgM: VlsE; IgG: (VlsE)	+ (B31, IP90) - (PT7)	66	x		x	x	x	
Lyme13	1 month Erythema migrans + other symptoms	IgM: OspC; IgG: VlsE	+	73	x			x	x	
Lyme15	2 days Erythema migrans + other symptoms	IgG: (VlsE)	-	29	x					
Lyme16	0-4 days Erythema migrans	Negative	-	53	x					
Tick1	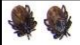 Tick bite 2 days ago	negative	-	64					x	
Tick2	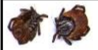 Tick bite 3 days ago	negative	-	73					x	
Tick3	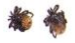 Recruitment on the day of tick bite	negative	-	71	x				x	
Tick4	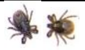 Tick bite 1 day ago (2012)	positive (IgM: OspC) > positive serology (~2006)	-	63	x					
Tick5	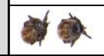 Tick bite 1 day ago (2012)	positive (IgM: OspC, IgG: VlsE and DbpA) > Lyme disease in 2004	+	64	x					
Comp ctrl	n.n.	n.n.	n.n.	59	x					
Healthy	n.n.	n.n.	n.n.	66	x					
Healthy	n.n.	n.n.	n.n.	46	x					
Healthy	n.n.	n.n.	n.n.	51	x					
Healthy	n.n.	n.n.	n.n.	69	x					
Healthy	n.n.	n.n.	n.n.	54	x					
Healthy	n.n.	negative	-	65				x	x	
Healthy	n.n.	negative	-	55				x	x	
Healthy	n.n.	negative	-	31					x	
Healthy	n.n.	negative	-	30				x	x	
Healthy (seronegative)	n.n.	negative	-	51			x	x	x	
Healthy (seronegative)	n.n.	negative	-	59				x	x	
Healthy (seronegative)	n.n.	negative	-	50				x	x	
Healthy	n.n.	negative	-	59					x	
seropositive	n.n.	n.n.	n.n.	*		x	x			
seropositive	n.n.	n.n.	n.n.	*		x	x			
seropositive	n.n.	n.n.	n.n.	*		x	x			
seropositive	n.n.	n.n.	n.n.	1961		x (IP90)				
seropositive	n.n.	n.n.	n.n.	1960		x (IP90)				
seropositive	n.n.	n.n.	n.n.	1965		x (IP90)				
seronegative	n.n.	n.n.	n.n.	1961		x (IP90)				
seronegative	n.n.	n.n.	n.n.	1960		x (IP90)				
seronegative	n.n.	n.n.	n.n.	1965		x (IP90)				
seropositive	n.n.	n.n.	n.n.	*		x				
seronegative	n.n.	n.n.	n.n.	*		x				
seropositive	n.n.	n.n.	n.n.	*		x				
seronegative	n.n.	n.n.	n.n.	*		x				
seropositive	n.n.	n.n.	n.n.	*		x				
Healthy	n.n.	n.n.	n.n.	60				x		
Healthy	n.n.	n.n.	n.n.	*				x		
Healthy	n.n.	n.n.	n.n.	*				x		

Supplementary Table 2

SUPPLEMENTARY TABLE 2 Primer sequences.

**Primers deep sequencing**	
65pA-MID21-8N-4-8N-M-Quake	GCGTGTCTCCGACTCAGTCGCAATTACNNNNNNNNGACTNNNNNNNNGGGAATTCTCACAGGAGACG
67pA-MID9-8N-4-8N-M-Quake	GCGTGTCTCCGACTCAGTGAGCGGAACNNNNNNNNGACTNNNNNNNNGGGAATTCTCACAGGAGACG
68pA-MID15-8N-4-8N-M-Quake	GCGTGTCTCCGACTCAGTCTAGAGGTCNNNNNNNNGACTNNNNNNNNGGGAATTCTCACAGGAGACG
69pA-MID23-8N-4-8N-M-Quake	GCGTGTCTCCGACTCAGTGCCACGAACNNNNNNNNGACTNNNNNNNNGGGAATTCTCACAGGAGACG
70pA-MID22-8N-4-8N-M-Quake	GCGTGTCTCCGACTCAGTTCGAGACGCNNNNNNNNGACTNNNNNNNNGGGAATTCTCACAGGAGACG
71pA-MID25-8N-4-8N-M-Quake	GCGTGTCTCCGACTCAGCCTGAGATACNNNNNNNNGACTNNNNNNNNGGGAATTCTCACAGGAGACG
59pA-MID9-8N-4-8N-G-Quake	GCGTGTCTCCGACTCAGTGAGCGGAACNNNNNNNNGACTNNNNNNNNAAGACCGATGGGCCCTTG
60pA-MID15-8N-4-8N-G-Quake	GCGTGTCTCCGACTCAGTCTAGAGGTCNNNNNNNNGACTNNNNNNNNAAGACCGATGGGCCCTTG
61pA-MID21-8N-4-8N-G-Quake	GCGTGTCTCCGACTCAGTCGCAATTACNNNNNNNNGACTNNNNNNNNAAGACCGATGGGCCCTTG
62pA-MID23-8N-4-8N-G-Quake	GCGTGTCTCCGACTCAGTGCCACGAACNNNNNNNNGACTNNNNNNNNAAGACCGATGGGCCCTTG
63pA-MID22-8N-4-8N-G-Quake	GCGTGTCTCCGACTCAGTTCGAGACGCNNNNNNNNGACTNNNNNNNNAAGACCGATGGGCCCTTG
64pA-MID25-8N-4-8N-G-Quake	GCGTGTCTCCGACTCAGCCTGAGATACNNNNNNNNGACTNNNNNNNNAAGACCGATGGGCCCTTG
52LpP1-V1-FR2-BIO	CTATGGGCAGTCGGTGATCTGGGTGCGACAGGCCCCTGGACAA
53LpP1-V2-FR2_BIO	CTATGGGCAGTCGGTGATTGGATCCGTCAGCCCCCAGGGAAGG
54LpP1-V3-FR2-BIO	CTATGGGCAGTCGGTGATGGTCCGCCAGGCTCCAGGGAA
55LpP1-V4-FR2-BIO	CTATGGGCAGTCGGTGATTGGATCCGCCAGCCCCCAGGGAAGG
56LpP1-V5-FR2-BIO	CTATGGGCAGTCGGTGATGGGTGCGCCAGATGCCCGGGAAAGG
57LpP1-V6-FR2-BIO	CTATGGGCAGTCGGTGATTGGATCAGGCAGTCCCCATCGAGAG
58LpP1-V7-FR2-BIO	CTATGGGCAGTCGGTGATTTGGGTGCGACAGGCCCCTGGACAA
amp_A	CCATCTCATCCCTGCGTGTCTCCGACTCAG
amp_P1	CCTCTCTATGGGCAGTCGGTGAT
**Primers single cell PCRs**	
Single cell 1 - VHL-1	TCACCATGGACTGSACCTGGA
Single cell 1 - VHL-2	CCATGGACACACTTTGYTCCAC
Single cell 1 - VHL-3	TCACCATGGAGTTTGGGCTGAGC
Single cell 1 - VHL-4	AGAACATGAAACAYCTGTGGTTCTT
Single cell 1 - VHL-5	ATGGGGTCAACCGCCATCCT
Single cell 1 - VHL-6	ACAATGTCTGTCTCCTTCCTCAT
Single cell 1 - CμII	CAGGAGACGAGGGGGAAAAG
Single cell 1 - CγII	GCCAGGGGGAAGACSGATG
Single cell 2 - CαII	GCTCAGCGGGAAGACCTT
Single cell 2 - VH-1 - nr	CAGGTSCAGCTGGTRCAGTC
Single cell 2 - VH-2 - nr	CAGRTCACCTTGAAGGAGTC
Single cell 2 - VH-3 - nr	SAGGTGCAGCTGGTGGAGTC
Single cell 2 - VH-4 - nr	CAGGTGCAGCTGCAGGAGTC
Single cell 2 - VH-5 - nr	GARGTGCAGCTGGTGCAGTC
Single cell 2 - VH-6 - nr	CAGGTACAGCTGCAGCAGTC
Single cell 2 - CμIII - nr	GAAAAGGGTTGGGGCGGATGC
Single cell 2 - CγIII - nr	GACSGATGGGCCCTTGGTGGA
Single cell 3 - CαIII - nr	GACCTTGGGGCTGGTCGGGGA
